# Silicon Nanoparticle Seed Priming Modulates Germination, Early Seedling Growth, and Biochemical Responses of Soybean Under PEG-Induced Drought Stress

**DOI:** 10.3390/plants15203226

**Published:** 2026-10-21

**Authors:** Nikolina Šimić, Dejan Agić, Sonja Grljušić, Zvonimir Zdunić, Luka Andrić, Irena Tomičić, Miroslav Lisjak, Manda Antunović, Ivana Varga

**Affiliations:** 1 Agricultural Institute Osijek, Južno Predgrađe 17, 31000 Osijek, Croatia; nikolina.simic@poljinos.hr (N.Š.); sonja.grljusic@poljinos.hr (S.G.); zvonimir.zdunic@poljinos.hr (Z.Z.); luka.andric@poljinos.hr (L.A.); 2 Faculty of Agrobiotechnical Sciences Osijek, Josip Juraj Strossmayer University of Osijek, Vladimira Preloga 1, 31000 Osijek, Croatia; irena.tomicic@fazos.hr (I.T.); miroslav.lisjak@fazos.hr (M.L.); manda.antunovic@fazos.hr (M.A.); ivana.varga@fazos.hr (I.V.)

**Keywords:** soybean (*Glycine max* (L.) Merr.), drought stress, seed nanopriming, silicon dioxide nanoparticles, germination, early seedling growth, biochemical responses

## Abstract

Drought stress during germination and early seedling growth can impair soybean performance, while silicon nanoparticle (SiNP) seed priming may modulate early stress responses. This study aimed to evaluate the effects of SiNP seed priming on the germination, early seedling growth, and biochemical responses of 11 soybean (*Glycine max* (L.) Merr.) genotypes under non-stressed conditions and two levels of PEG-induced drought stress (5% and 10% PEG 6000), and to assess how these responses varied with stress intensity and genotype. Seeds were untreated, hydroprimed, or primed with SiO_2_ nanoparticles at 500 or 1000 mg L^−1^. Across genotypes and stress levels, priming generally increased germination energy and total germination and reduced non-germinated seeds, with similar improvements observed following hydropriming and SiNP priming, whereas normal seedling development was not consistently improved. Increasing stress strongly inhibited shoot elongation and seedling fresh mass, whereas root growth was comparatively maintained. SiNP priming generally resulted in higher seedling fresh mass, with SiNPs 500 producing higher values than the untreated control in most genotypes under mild stress and all genotypes under severe stress. SiNP priming also increased proline accumulation and total amylolytic activity, with SiNPs 1000 showing the highest values under mild and severe stress. Overall, SiNP seed priming most consistently improved soybean germination under PEG-induced drought stress, whereas its effects on early seedling growth and biochemical responses varied with nanoparticle concentration, genotype, and stress intensity.

## 1. Introduction

Soybean (*Glycine max* (L.) Merr.) is one of the world’s most important grain legumes and a major source of plant-based protein and vegetable oil, with diverse applications in food, animal feed, and industry [1,2]. In addition to its nutritional and economic importance, soybean contributes to sustainable agricultural systems through its symbiotic association with nitrogen-fixing rhizobia, particularly *Bradyrhizobium* spp., which facilitates biological nitrogen fixation and can reduce reliance on synthetic nitrogen fertilizers [3,4,5]. However, maintaining stable soybean productivity is becoming increasingly challenging under changing climatic conditions, with drought and water deficit representing major environmental constraints to soybean growth and yield [6,7,8]. Germination and early seedling growth are particularly vulnerable to water deficit, as insufficient water availability can impair germination and early seedling growth, ultimately affecting crop stand establishment [9,10,11]. Germination involves a coordinated series of physiological and biochemical processes initiated by seed imbibition, including the reactivation of respiration, protein synthesis, reserve mobilization, and activation of hydrolytic enzymes [12,13,14]. Restricted water availability can impair the physiological and biochemical processes required for successful germination, resulting in delayed or reduced germination, decreased seedling vigor, impaired root development, and poor seedling growth. Because early root development is essential for water acquisition and early seedling growth under water-limited conditions, maintaining root growth and overall seedling performance during early development is critical for soybean resilience to water deficit [15]. Drought stress is also associated with disruption of cellular redox homeostasis and excessive accumulation of reactive oxygen species (ROS). Although ROS function as important signalling molecules involved in stress perception and acclimation, their excessive accumulation can cause oxidative damage to membrane lipids, proteins, and nucleic acids. Plants counteract oxidative stress through coordinated enzymatic and non-enzymatic antioxidant systems, including antioxidant enzymes, phenolic compounds, flavonoids, carotenoids, and compatible osmolytes such as proline. Under severe or prolonged drought, however, endogenous defence mechanisms may become insufficient to maintain cellular homeostasis [16,17,18].

Seed priming has been shown to improve germination and early seedling performance under abiotic stress, although the magnitude of the response may depend on stress intensity [19,20,21]. Seed priming involves controlled hydration of seeds to initiate pre-germinative metabolic and physiological processes while preventing radicle protrusion, followed by re-drying before sowing. This treatment can promote faster and more synchronized germination, enhance seedling vigor, and improve plant tolerance to abiotic stress [20]. Conventional seed-priming approaches include hydropriming, osmopriming, nutrient priming, hormonal priming, redox priming, chemical priming, solid matrix priming, and biopriming [22,23]. However, their effectiveness may vary depending on genotype, environmental conditions, and treatment characteristics [24]. More recently, nanopriming has emerged as an advanced seed-treatment approach in which nanoparticles are used as priming agents to enhance germination, early seedling growth, and tolerance to abiotic stress [25,26]. Nanoparticles possess unique physicochemical properties that can influence their uptake, transport, and interactions with plant tissues [19,27]. When applied as seed-priming agents, nanoparticles can influence germination, nutrient uptake, antioxidant responses, and stress-signalling pathways, thereby contributing to improved seedling performance and abiotic stress tolerance [28,29].

Among nanomaterials investigated in agriculture, silicon nanoparticles (SiNPs) are of particular interest because silicon is recognized as a beneficial element that can enhance plant tolerance to abiotic stress. Silicon has been associated with improved water relations, water-use efficiency, photosynthetic performance, structural protection, and antioxidant defence under drought conditions [30,31]. Recent experimental studies have demonstrated beneficial effects of silicon on soybean under water-limited conditions, including improved germination and seedling growth following silicon priming [32] and enhanced growth and root characteristics following soil-applied silicon [33], as well as improved physiological performance and yield following nanosilica application under drought stress [34].

The nanoscale form of silicon may provide additional advantages because of its greater specific surface area and potentially enhanced biological reactivity [27,35]. Evidence from other crop species suggests that SiO_2_ nanoparticle seed priming can enhance germination and early seedling development and improve plant responses to water deficit [36,37,38]. Beyond their effects on germination and early growth, SiNPs have also been shown to modulate physiological, biochemical, and redox responses associated with drought tolerance across a range of crop species, including wheat, rice, and cucumber [39,40,41]. Moreover, seed priming with silicon nanoparticles has been shown to improve drought-stress tolerance in soybean genotype DS 2614 by enhancing antioxidant defence and osmotic adjustment [42].

Despite growing interest in silicon-based nanotechnologies, the effects of SiNP seed priming on soybean germination and early seedling development remain insufficiently characterized across multiple genotypes and different levels of drought stress. In particular, it remains unclear how SiNP concentration, stress intensity, and genotype jointly influence germination, early seedling growth, and associated biochemical responses.

Therefore, this study aimed to evaluate the effects of SiNP seed priming on the germination, early seedling growth, and biochemical responses of 11 soybean genotypes exposed to increasing levels of PEG-induced drought stress, and to determine whether these responses depend on SiNP concentration, drought intensity, and genotype.

## 2. Results

### 2.1. Germination Traits

The three-way analysis of variance revealed highly significant effects of seed treatment (T), stress level (PEG), and genotype (G) on all evaluated germination traits. Significant T × PEG, T × G, PEG × G, and T × PEG × G interactions were also detected (*p* ≤ 0.001) (Table 1).

**Figure 1:**
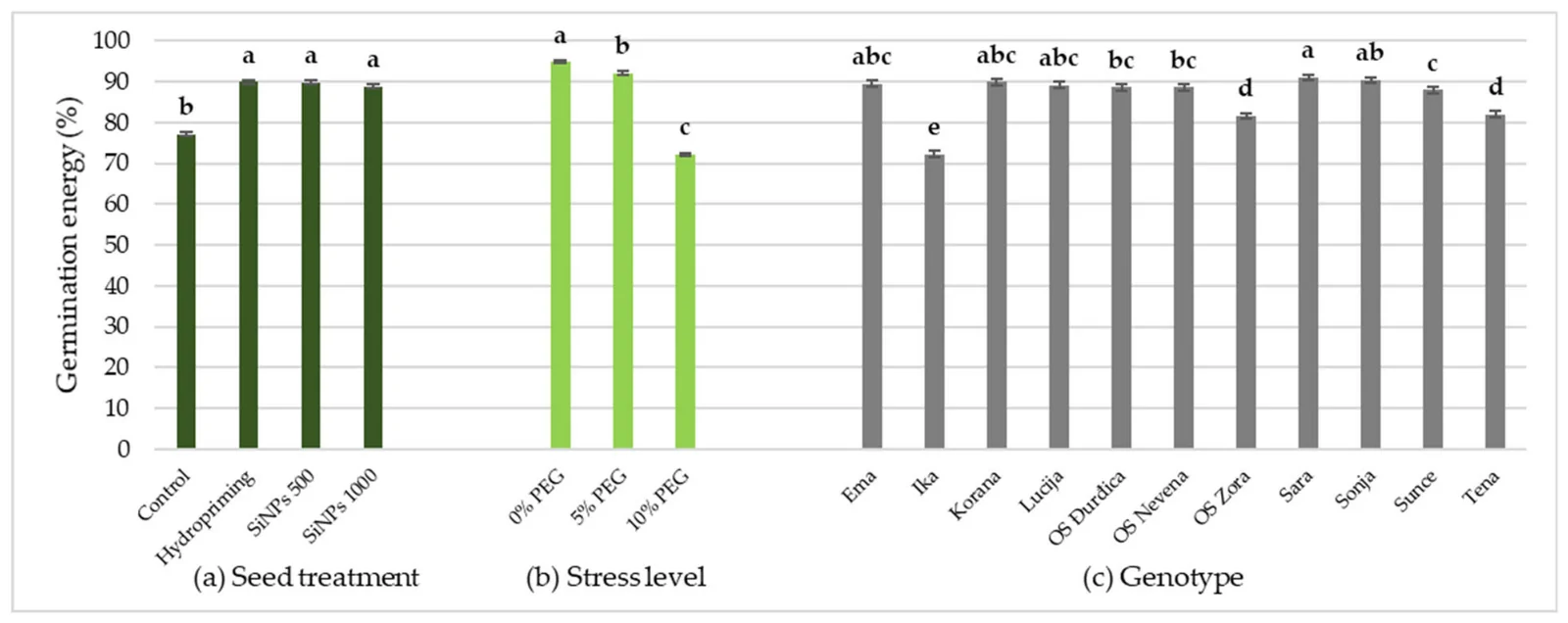
Germination energy (%) as affected by (**a**) seed treatment, (**b**) drought stress level, and (**c**) genotype. In (**a**), values represent means across 11 soybean genotypes and three drought stress levels; in (**b**), values represent means across 11 soybean genotypes and all four seed treatments; and in (**c**), values represent means across all four seed treatments and three drought stress levels. Bars represent mean values ± SE. Different letters indicate statistically significant differences among means within each panel (LSD test, *p* ≤ 0.05).

Clear differences in germination energy were observed among seed treatments, stress levels, and genotypes (Figure 1a–c). Hydropriming (90.0%), SiNPs 500 (89.8%), and SiNPs 1000 (88.9%) resulted in significantly higher germination energy than the untreated control (77.2%), with no significant differences among the three priming treatments (Figure 1a). Increasing PEG concentration progressively reduced germination energy, from 95.0% under non-stressed conditions to 92.1% at 5% PEG and 72.2% at 10% PEG, corresponding to a 24.0% reduction under severe stress compared with the non-stressed conditions (Figure 1b). Genotypic differences were also evident, with germination energy ranging from 72.2% in *Ika* to 91.0% in *Sara* (Figure 1c).

**Figure 2:**
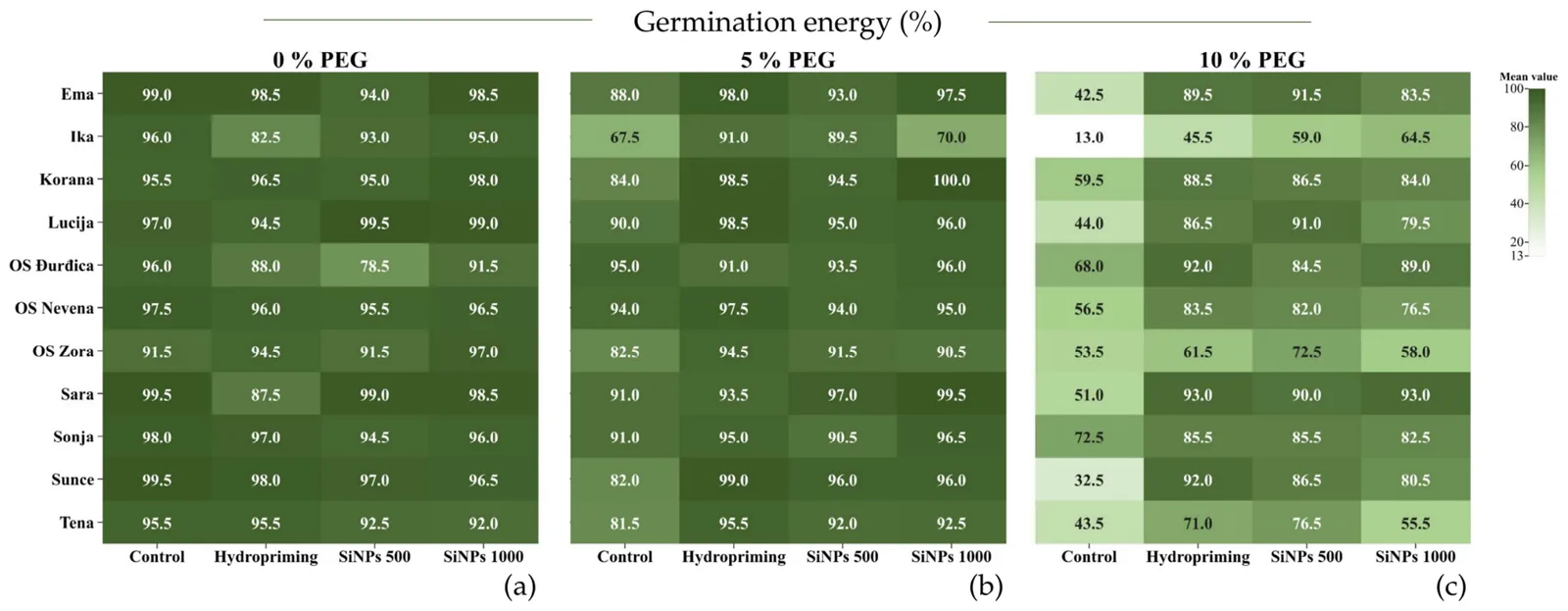
Interaction between soybean genotype and seed treatment for germination energy (%) under (**a**) non-stressed conditions (0% PEG), (**b**) mild drought stress (5% PEG), and (**c**) severe drought stress (10% PEG). Values represent mean germination energy.

The significant T × PEG × G interaction (*p* < 0.0001) demonstrated that the effect of seed treatment depended strongly on both genotype and stress intensity (Figure 2a–c). Under mild stress, the response was treatment-dependent; for example, in *Ika*, germination energy increased from 67.5% in the untreated control to 89.5% with SiNPs 500 and 91.0% with hydropriming, whereas SiNPs 1000 resulted in 70.0%. Under severe stress, the differences became more pronounced, particularly in *Ika*, where SiNPs 1000 increased germination energy from 13.0% in the untreated control to 64.5%. SiNPs 500 showed the highest germination energy under severe stress in *Ema* (91.5%) and *Lucija* (91.0%), highlighting the genotype-dependent response to SiNP treatments under increasing drought stress.

**Figure 3:**
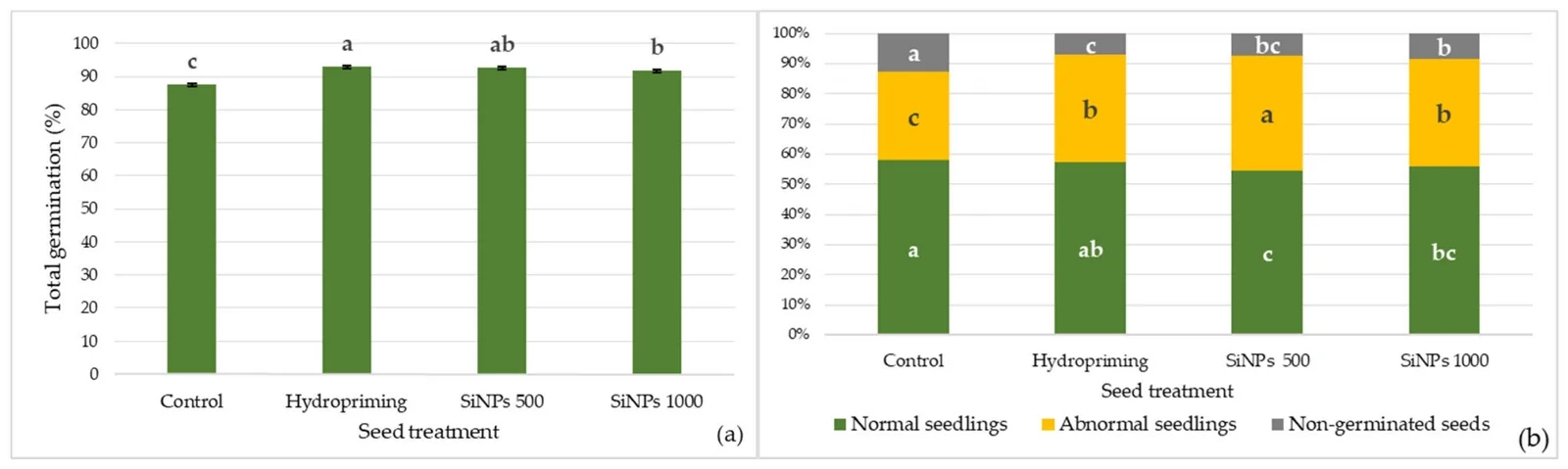
Effects of seed treatment on (**a**) total germination (%) and (**b**) proportional distribution (100%) of normal seedlings, abnormal seedlings, and non-germinated seeds. Values represent means across 11 soybean genotypes and three drought stress levels. Bars represent mean values. Different lowercase letters indicate statistically significant differences among seed treatment means within each parameter (LSD test, *p* ≤ 0.05).

Total germination (TG) differed among seed treatments, with hydropriming (92.9%), SiNPs 500 (92.6%), and SiNPs 1000 (91.7%) resulting in significantly higher values than the untreated control (87.5%) (Figure 3a). The increase in total germination was accompanied by a reduction in the proportion of non-germinated seeds, from 12.5% in the untreated control to 7.1% with hydropriming, 7.4% with SiNPs 500, and 8.3% with SiNPs 1000 (Figure 3b). In contrast, the proportion of normal seedlings was highest in the untreated control (58.0%), which did not differ significantly from hydropriming (57.5%), whereas SiNPs 500 resulted in a significantly lower proportion of normal seedlings (54.6%). The proportion of abnormal seedlings ranged from 29.5% in the untreated control to 38.0% with SiNPs 500, with hydropriming (35.4%) and SiNPs 1000 (35.8%) showing intermediate values. Thus, although seed priming increased total germination primarily through a reduction in non-germinated seeds, this was not accompanied by an increase in the proportion of normal seedlings.

While the overall effects of seed treatment and drought stress were significant, the proportion of normal seedlings also varied markedly among genotypes, as indicated by the significant three-way interaction among seed treatment, drought stress, and genotype (*p* < 0.0001). Under non-stressed conditions (Figure S1a), the highest proportion of normal seedlings was recorded in *Ika* in the untreated control (89.0%), and the lowest proportion was recorded in *Sara* with hydropriming (33.5%). At mild drought stress (5% PEG) (Figure S1b), genotype-dependent responses became more pronounced. SiNPs 1000 increased the proportion of normal seedlings in *Korana* from 54.5% in the untreated control to 83.5%. Under severe drought stress (10% PEG) (Figure S1c), the proportion of normal seedlings declined further, but priming maintained higher values in several genotypes. The most pronounced responses were observed in *Ika*, where the proportion of normal seedlings increased from 11.5% in the untreated control to 40.0% with SiNPs 1000.

**Figure 4:**
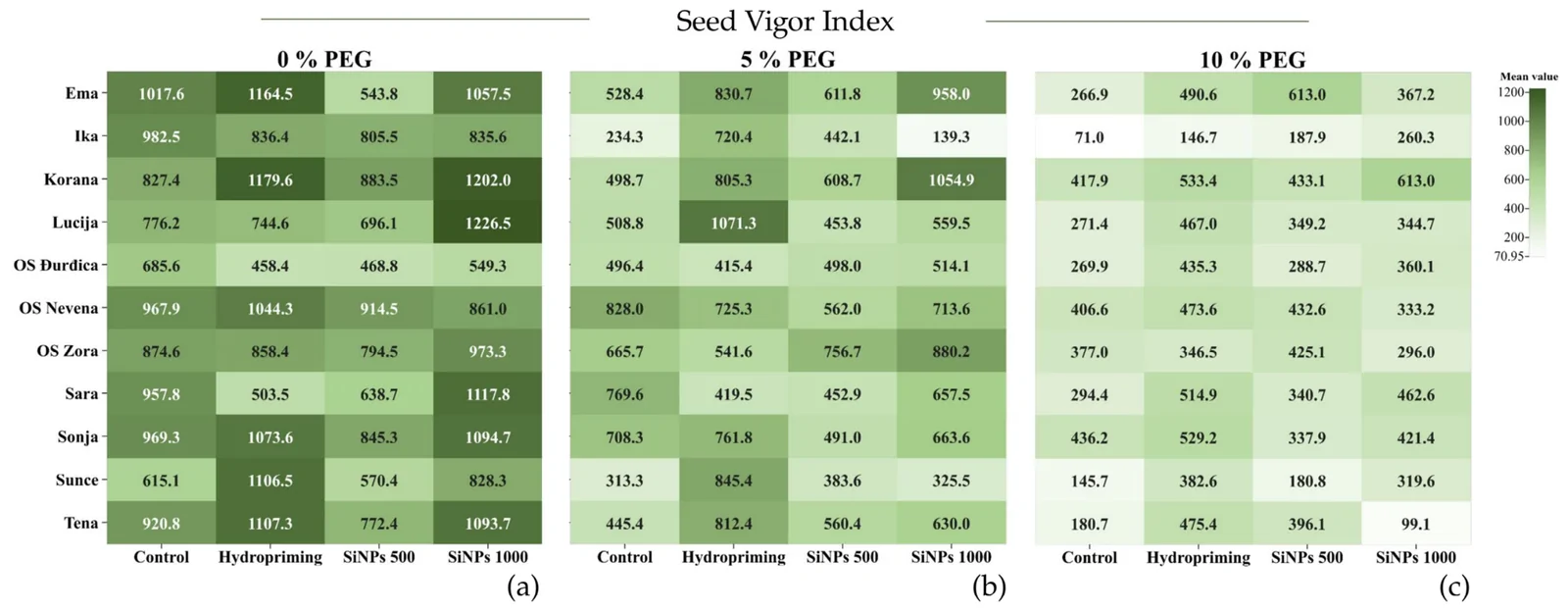
Interaction between soybean genotype and seed treatment for seed vigor index (SVI) under (**a**) non-stress conditions (0% PEG), (**b**) mild drought stress (5% PEG), and (**c**) severe drought stress (10% PEG). Values represent mean SVI values.

The seed vigor index (SVI) was particularly strongly affected by drought stress and genotype, while the response to seed treatment varied among genotypes and stress levels. Under non-stressed conditions (Figure 4a), SiNPs 1000 resulted in particularly high SVI in *Lucija* (1226.5) and *Korana* (1202.0). At mild drought stress (5% PEG) (Figure 4b), hydropriming markedly increased SVI in *Lucija* (1071.3), while SiNPs 1000 resulted in high values in *Korana* (1054.9), *Ema* (958.0), and *OS Zora* (880.2). Priming maintained higher SVI in several genotypes under severe stress (Figure 4c). In particular, SiNPs 1000 increased SVI in *Korana* from 417.9 in the untreated control to 613.0, while in *Ika*, the corresponding increase was from 71.0 to 260.3. In *Ema*, SiNPs 500 resulted in 613.0 compared with 266.9 in the control. However, the response was not consistent across genotypes, as demonstrated by *Tena*, where SiNPs 1000 resulted in a lower SVI than the untreated control (99.1 vs. 180.7).

### 2.2. Early Seedling Growth and Morphological Responses

The effects observed for germination traits were accompanied by significant changes in early seedling growth and morphology. All examined morphological parameters were significantly affected by genotype, stress level, and seed treatment, with significant two- and three-way interactions among these factors (Table 2).

**Figure 5:**
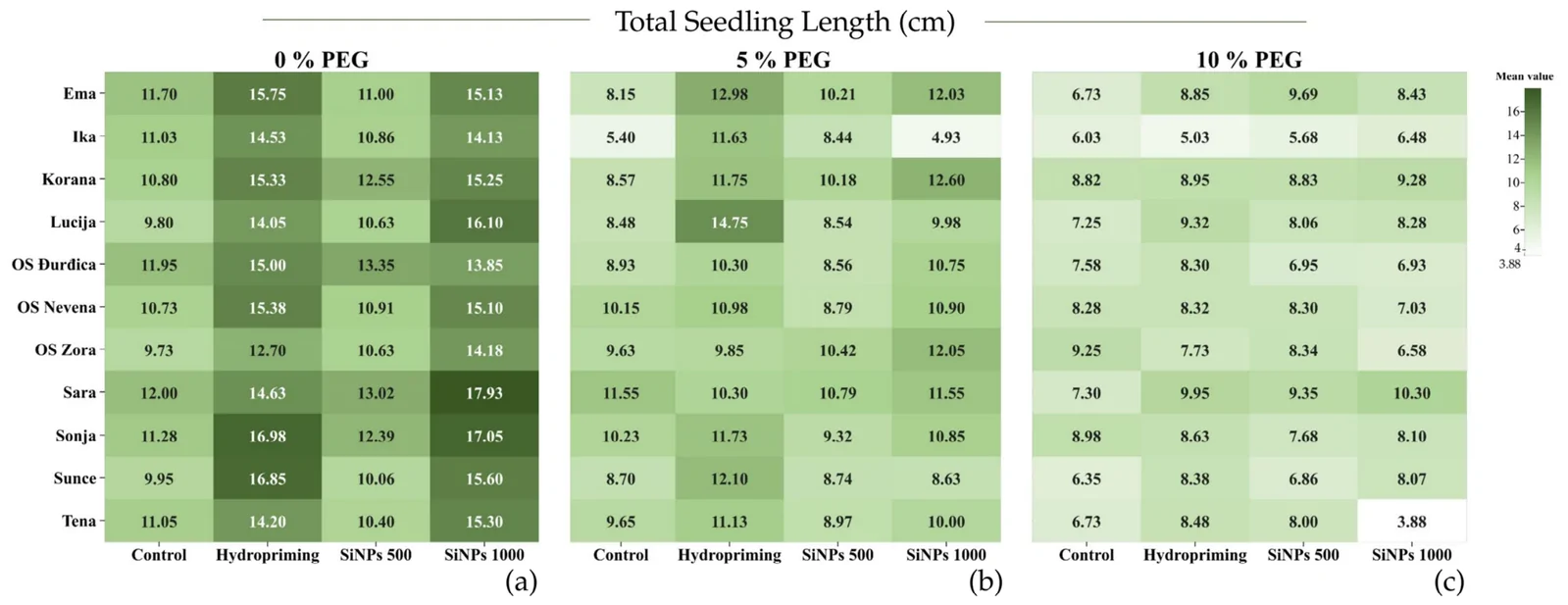
Interaction between soybean genotype and seed treatment for total seedling length (TSL) (cm) under (**a**) non-stressed conditions (0% PEG), (**b**) mild drought stress (5% PEG), and (**c**) severe drought stress (10% PEG). Values represent mean total seedling length.

Total seedling length (TSL) decreased progressively with increasing PEG concentration, although the magnitude of this response varied considerably among genotypes and seed treatments (Figure 5a–c). Under non-stressed conditions (0% PEG), seedlings treated with hydropriming and SiNPs 1000 tended to show higher total seedling length than those in the untreated control and SiNPs 500. The highest value was recorded in *Sara* following SiNPs 1000 treatment (17.93 cm). Under mild drought stress (5% PEG), total seedling length generally decreased, although the extent of the reduction varied among genotypes and seed treatments. Notably, hydropriming maintained a high value in *Lucija* (14.75 cm), while SiNPs 1000 resulted in relatively high values in *Korana* (12.60 cm) and *OS Zora* (12.05 cm). Under severe drought stress (10% PEG), the contrasting responses among genotypes and treatments became more evident. *Sara* maintained the highest seedling length with SiNPs 1000 (10.30 cm), whereas *Tena* showed a pronounced reduction under SiNPs 1000, from 15.30 cm at 0% PEG to 3.88 cm at 10% PEG (74.6%). In contrast, SiNPs 500 resulted in a comparatively small reduction in *Ema*, from 11.00 to 9.69 cm (11.9%). Overall, increasing drought intensity reduced total seedling length, although the magnitude of this reduction varied markedly among genotypes and seed treatments.

**Figure 6:**
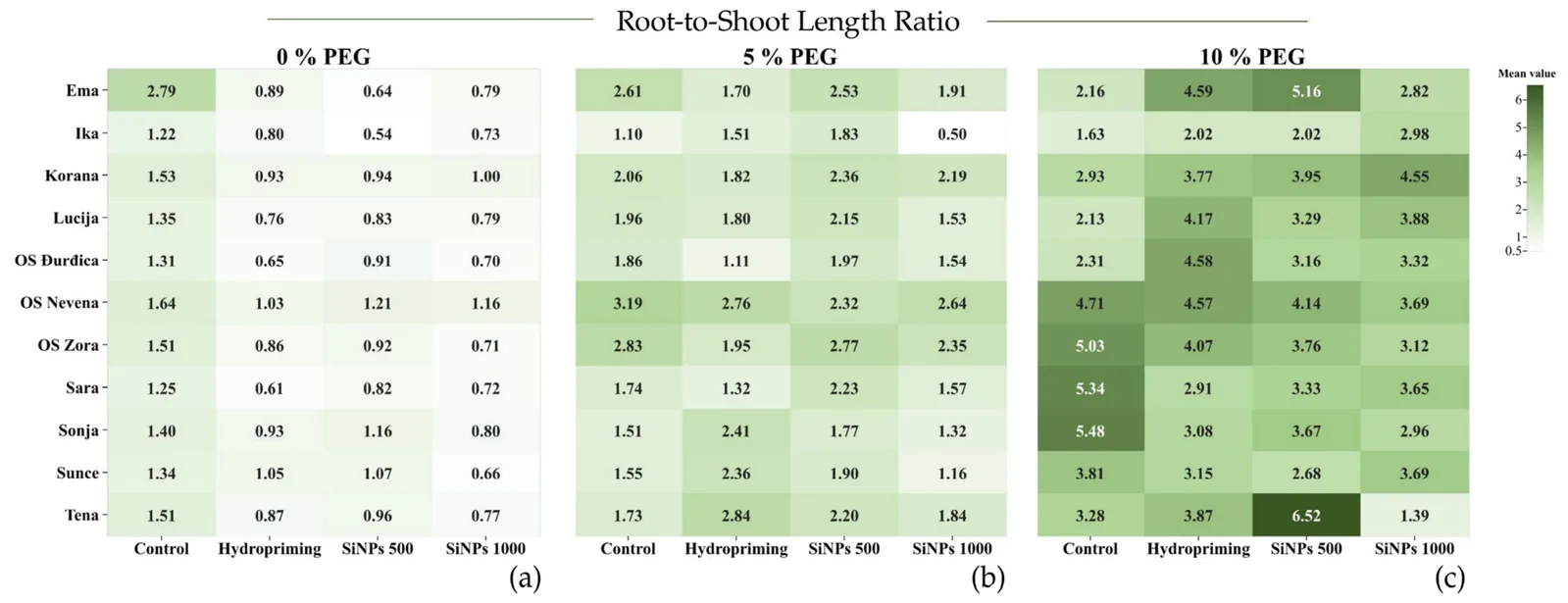
Interaction between soybean genotype and seed treatment for root-to-shoot length ratio (R/S) under (**a**) non-stressed conditions (0% PEG), (**b**) mild drought stress (5% PEG), and (**c**) severe drought stress (10% PEG). Values represent mean root-to-shoot length ratio.

Root (RL) and shoot length (SL), seedling fresh mass (SFM), and the root-to-shoot length ratio (R/S) differed in their responses to increasing drought stress. RL varied less markedly across PEG levels than SL, whereas shoot length exhibited a pronounced decline with increasing drought stress. Under non-stressed conditions, hydropriming and SiNPs 1000 produced the longest shoots (8.15 and 8.64 cm, respectively), while SL was substantially reduced at 10% PEG across all treatments (Figure S2a). In contrast, RL (Figure S2b) was less affected, with some treatments showing slightly higher values under mild drought stress. This differential response of root and shoot growth resulted in a pronounced shift in their relative contributions to total seedling length. At 0% PEG, root proportion (R/TSL) (Figure S2c) ranged from 44.0% with SiNPs 1000 to 59.4% in the untreated control, whereas at 10% PEG, it increased to 75.2–77.5% across treatments. Correspondingly, shoot proportion (S/TSL) decreased with increasing PEG concentration, from 40.6–56.0% at 0% PEG to only 22.5–24.9% at 10% PEG (Figure S2d). Accordingly, the root-to-shoot length ratio (R/S) generally increased with increasing drought stress, although the magnitude of the response varied considerably among genotypes and seed treatments (Figure 6). Under non-stressed conditions, the highest R/S was observed in *Ema* under the untreated control (2.79), while all priming treatments resulted in lower values. Under mild drought stress, responses to priming became more heterogeneous; for example, SiNPs 500 increased the R/S in *Ika* from 1.10 in the control to 1.83, representing a 66.4% increase. Under severe drought stress, both SiNP treatments showed higher R/S than the untreated control in five genotypes (*Ema*, *Ika*, *Korana*, *Lucija*, and *OS Đurđica*). The most pronounced response was observed in *Ema*, where the R/S increased from 2.16 in the control to 5.16 and 2.82 following SiNPs 500 and SiNPs 1000 treatments, respectively, while in *OS Đurđica*, it increased from 2.31 to 3.16 and 3.32, respectively.

**Figure 7:**
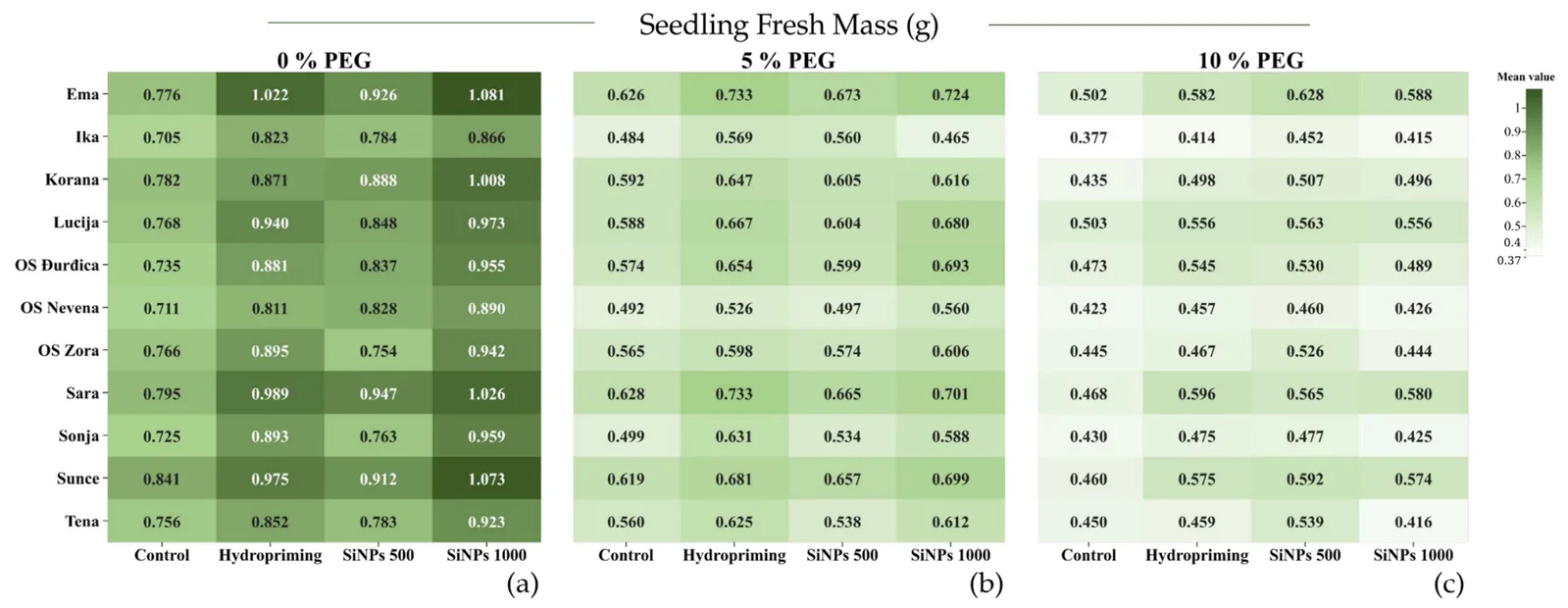
Interaction between soybean genotype and seed priming treatment for seedling fresh mass (SFM) (g) under (**a**) non-stressed conditions (0% PEG), (**b**) mild drought stress (5% PEG), and (**c**) severe drought stress (10% PEG). Values represent mean seedling fresh mass.

Seedling fresh mass (SFM) showed changes broadly consistent with those observed for seedling growth traits. SFM progressively decreased with increasing PEG concentration, although the magnitude of the reduction depended on genotype and seed treatment (Figure 7a–c). Under non-stressed conditions, priming generally resulted in higher SFM than the untreated control, with SiNPs 1000 producing the highest values in several genotypes, including *Ema* (1.081 g), *Sunce* (1.073 g), *Sara* (1.026 g), and *Korana* (1.008 g). At 5% PEG, SFM declined across all genotypes, while treatment responses became more variable. Under 10% PEG, priming treatments generally showed higher SFM than the untreated control in most genotypes, although the magnitude of the response varied among genotypes and SiNP concentrations. For example, SiNPs 500 resulted in higher SFM than the control in *Ema* (0.628 vs. 0.502 g) and *Tena* (0.539 vs. 0.450 g), whereas SiNPs 1000 reduced SFM in *Tena* to 0.416 g.

**Figure 8:**
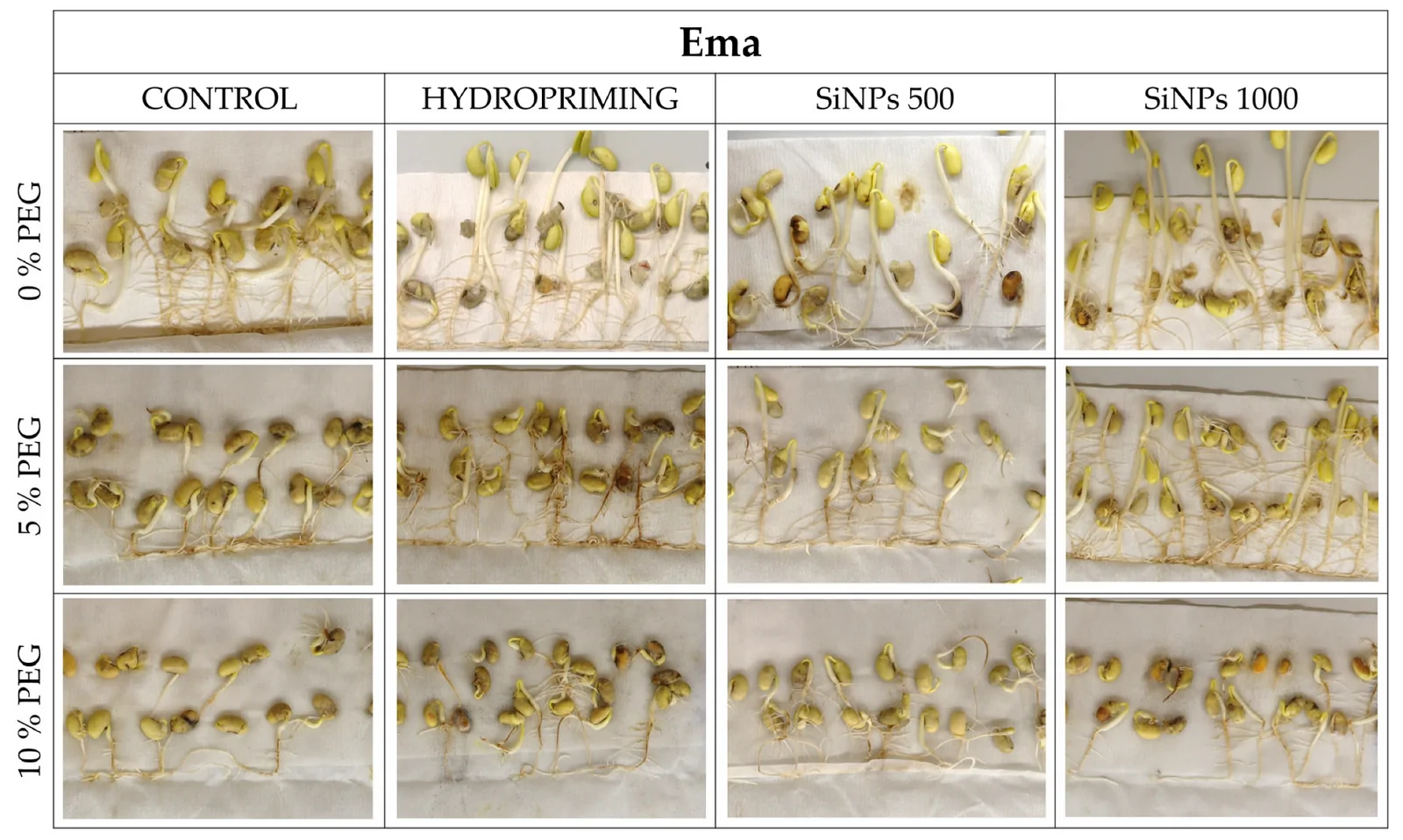

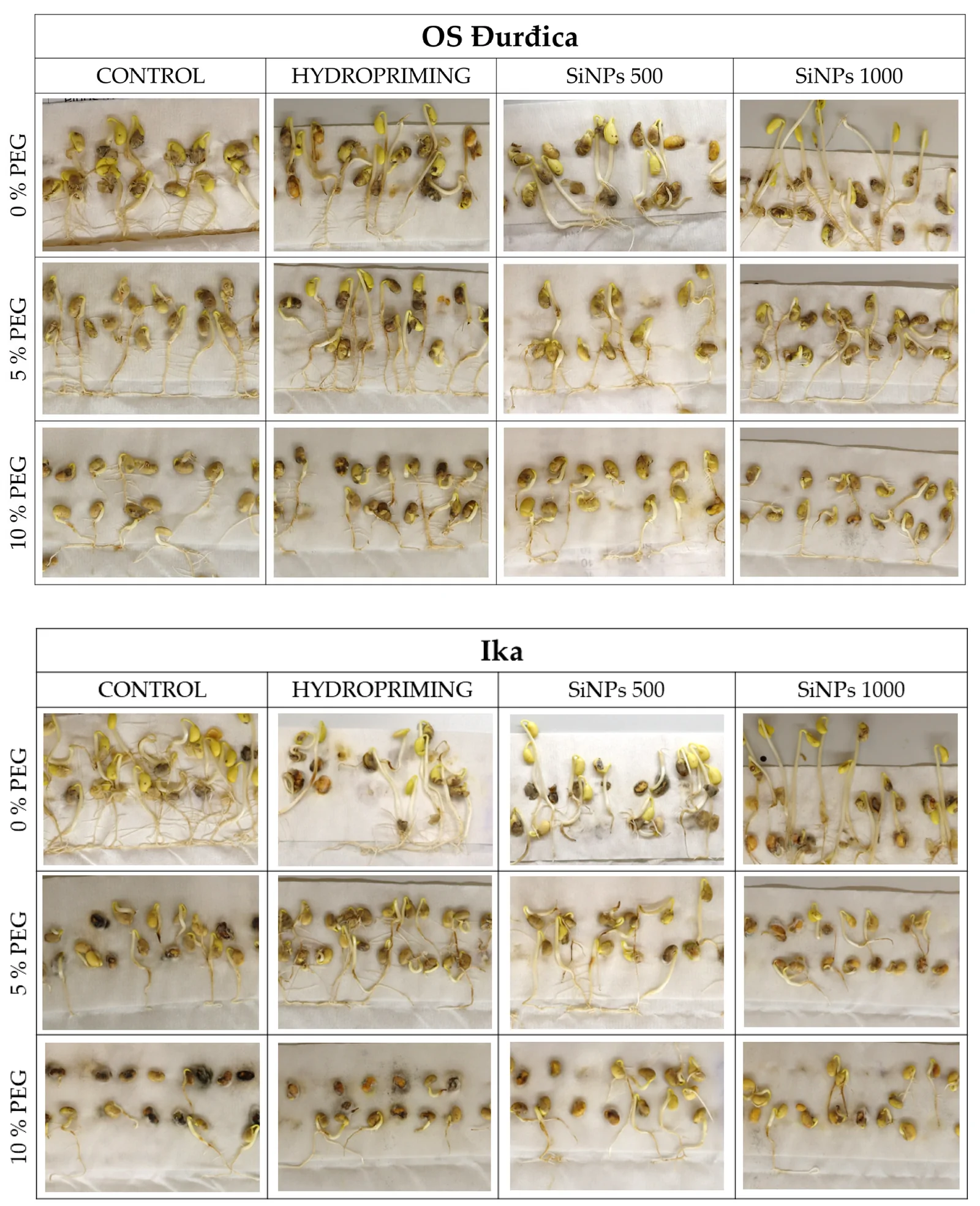
Representative soybean seedlings under different seed treatments and drought stress levels.

Overall, increasing drought stress strongly restricted shoot elongation and reduced seedling fresh mass, whereas root growth was comparatively maintained, resulting in a greater relative contribution of roots to total seedling length. However, the magnitude of these changes varied among seed treatments, with additional variation observed among genotypes. Representative soybean seedlings illustrating these morphological responses under different seed treatments and drought stress levels are shown in Figure 8.

### 2.3. Biochemical Responses

Biochemical traits responded differently to seed treatment, drought stress, and genotype. Most traits showed significant effects of the main factors and their interactions, including significant T × PEG × G interactions (*p* < 0.0001). Antioxidant capacity was the only trait that showed no significant interactions involving genotype, although significant effects of seed treatment, stress level, and their interaction were observed (Table 3).

#### 2.3.1. Proline and Antioxidant-Related Biochemical Responses

**Figure 9:**
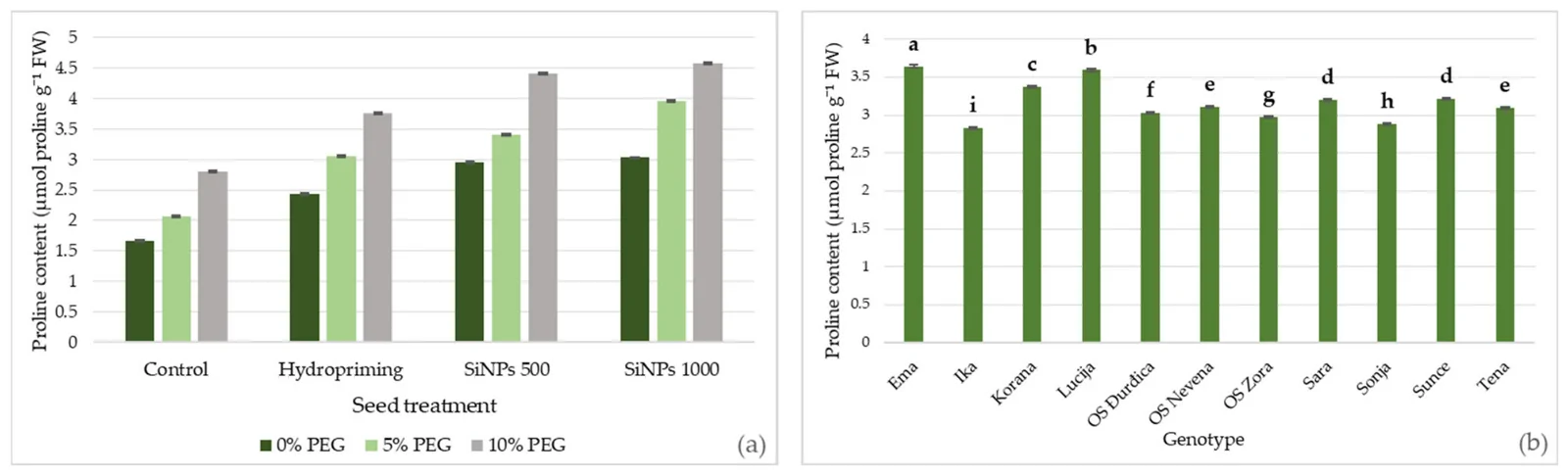
Effects of (**a**) seed treatment under different drought stress levels and (**b**) genotype on proline content. In (**a**), values represent means across 11 soybean genotypes for each combination of seed treatment and drought stress level. In (**b**), values represent genotype means across all four seed treatments and three drought stress levels. Bars represent mean values ± SE. Different letters in (**b**) indicate statistically significant differences among genotypes according to the LSD test (*p* ≤ 0.05).

Proline content increased with increasing PEG concentration across all seed treatments, although the magnitude of the response varied among treatments (Figure 9a). Across stress levels, the untreated control consistently showed the lowest proline content, increasing from 1.665 µmol g^−1^ FW at 0% PEG to 2.801 µmol g^−1^ FW at 10% PEG. Priming treatments showed higher values, with SiNPs 1000 generally showing the highest proline content, reaching 4.579 µmol g^−1^ FW under severe drought stress, followed by SiNPs 500 (4.414 µmol g^−1^ FW) and hydropriming (3.751 µmol g^−1^ FW). Proline content also differed significantly among genotypes (Figure 9b), ranging from 2.824 µmol g^−1^ FW in *Ika* to 3.643 µmol g^−1^ FW in *Ema*. *Ema* showed the highest mean proline content, followed by *Lucija* (3.593 µmol g^−1^ FW) and *Korana* (3.371 µmol g^−1^ FW), whereas *Ika* exhibited the lowest value.

**Figure 10:**
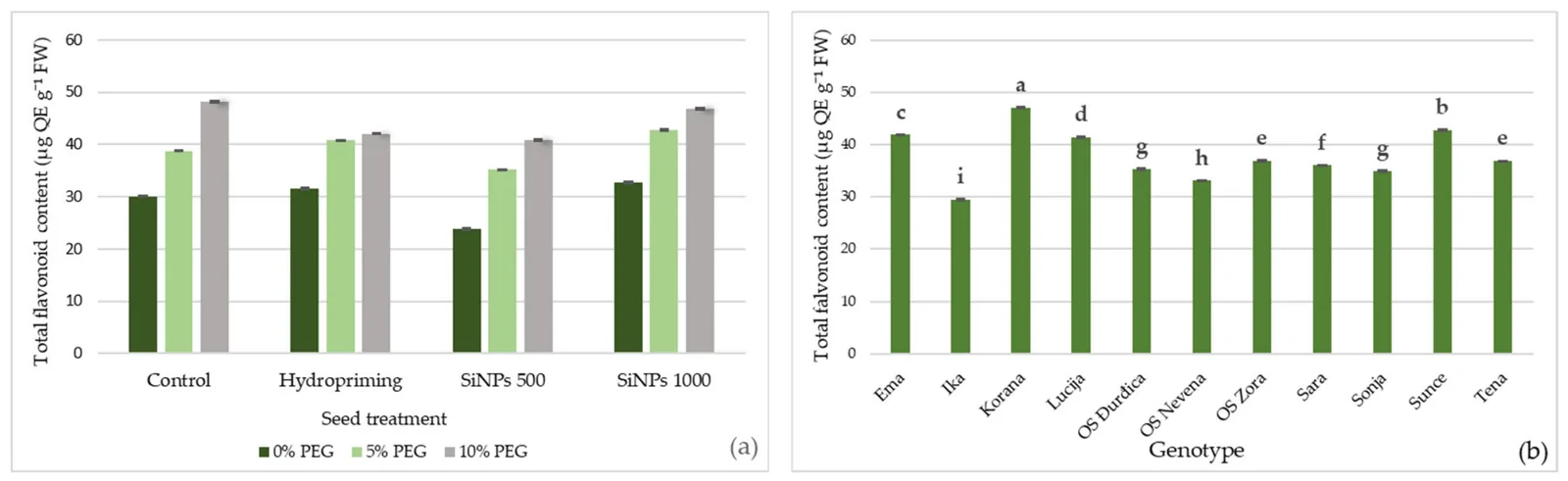
Effects of (**a**) seed treatment under different drought stress levels and (**b**) genotype on total flavonoid content. In (**a**), values represent means across 11 soybean genotypes for each combination of seed treatment and drought stress level. In (**b**), values represent genotype means across all four seed treatments and three drought stress levels. Bars represent mean values ± SE. Different letters in (**b**) indicate statistically significant differences among genotypes according to the LSD test (*p* ≤ 0.05).

Increasing PEG concentration was generally associated with higher total flavonoid content, with treatment-specific differences in the response (Figure 10a). In the untreated control, values increased from 30.088 µg QE g^−1^ FW at 0% PEG to 48.176 µg QE g^−1^ FW at 10% PEG, representing the highest total flavonoid content recorded under severe drought stress. Among the priming treatments, SiNPs 1000 showed the highest values at both 5 and 10% PEG (42.734 and 46.841 µg QE g^−1^ FW, respectively). Genotypic differences were also pronounced (Figure 10b), with mean flavonoid content varying from 29.465 µg QE g^−1^ FW in *Ika* to 47.037 µg QE g^−1^ FW in *Korana*.

**Figure 11:**
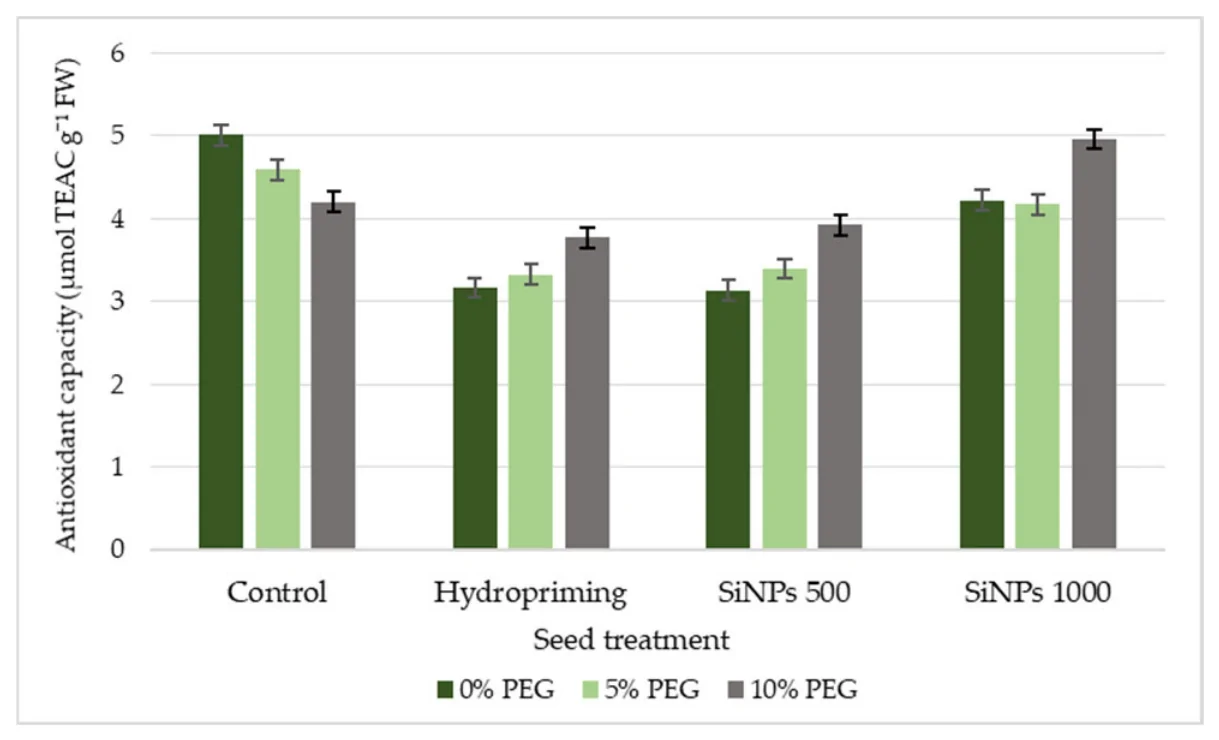
Effects of seed treatment on antioxidant capacity (AC) (µmol TEAC g^−1^ FW) under different drought stress levels. Values represent means across 11 soybean genotypes for each combination of seed treatment and drought stress level. Bars represent mean values ± SE.

Antioxidant capacity (AC) varied considerably across seed treatments and stress levels (Figure 11). Under non-stressed conditions, the highest value was recorded in the untreated control (5.01 µmol TEAC g^−1^ FW), whereas hydropriming and SiNPs 500 showed lower values (3.17 and 3.14 µmol TEAC g^−1^ FW, respectively). At 5% PEG, differences among treatments were less pronounced, with values ranging from 3.32 to 4.59 µmol TEAC g^−1^ FW. Under 10% PEG, the highest antioxidant capacity among the treatments was observed following SiNPs 1000. Antioxidant capacity was significantly affected by seed treatment and stress level; however, unlike the other biochemical parameters, no significant interactions involving genotype were detected (Table 3).

**Figure 12:**
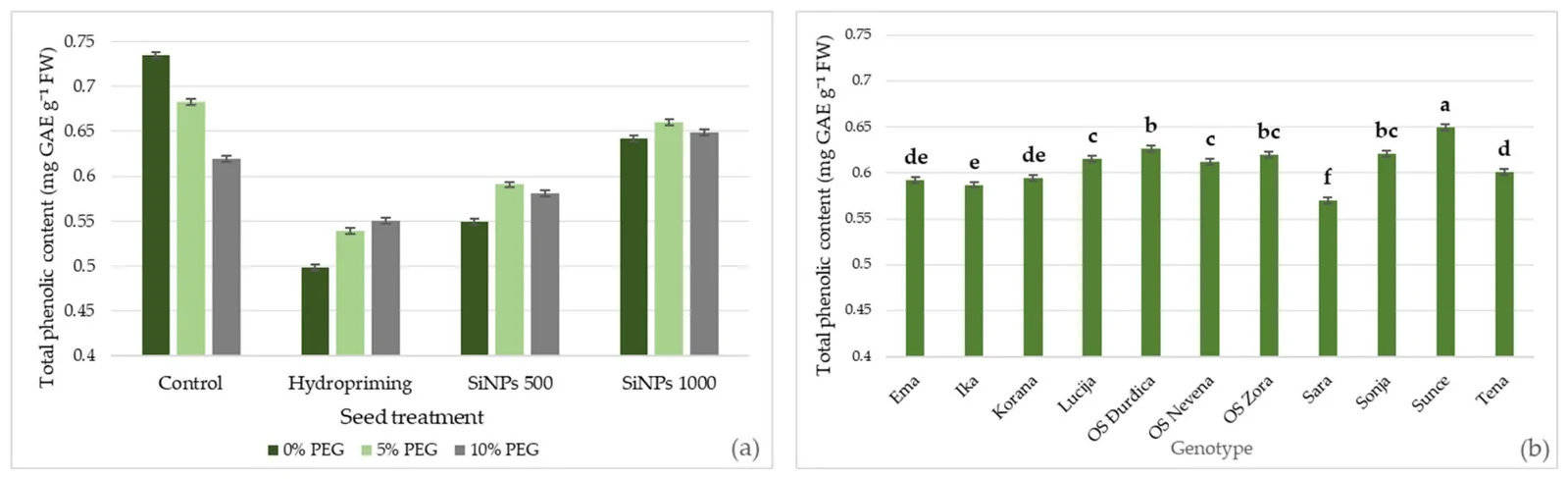
Effects of (**a**) seed treatment under different drought stress levels and (**b**) genotype on total phenolic content. In (**a**), values represent means across 11 soybean genotypes for each combination of seed treatment and drought stress level. In (**b**), values represent genotype means across all four seed treatments and three drought stress levels. Bars represent mean values ± SE. Different letters in (**b**) indicate statistically significant differences among genotypes according to the LSD test (*p* ≤ 0.05).

Total phenolic content showed a treatment-dependent response across PEG levels (Figure 12a). In the untreated control, phenolic content decreased from 0.734 mg GAE g^−1^ FW at 0% PEG to 0.620 mg GAE g^−1^ FW at 10% PEG. In contrast, hydropriming increased phenolic content from 0.498 to 0.550 mg GAE g^−1^ FW with increasing PEG concentration, while SiNPs 500 showed an increase from 0.550 mg GAE g^−1^ FW at 0% PEG to 0.591 mg GAE g^−1^ FW at 5% PEG. SiNPs 1000 maintained relatively high phenolic content across all PEG levels (0.642–0.660 mg GAE g^−1^ FW) and, under severe drought stress, showed the highest value among the priming treatments (0.649 mg GAE g^−1^ FW), which was also higher than that of the untreated control (0.620 mg GAE g^−1^ FW). Total phenolic content also differed significantly among genotypes (Figure 12b), ranging from 0.570 mg GAE g^−1^ FW in *Sara* to 0.650 mg GAE g^−1^ FW in *Sunce*. *Sunce* showed the highest mean phenolic content, followed by *OS Đurđica* (0.626 mg GAE g^−1^ FW), whereas *Sara* exhibited the lowest value.

#### 2.3.2. Photosynthetic Pigment Content

**Figure 13:**
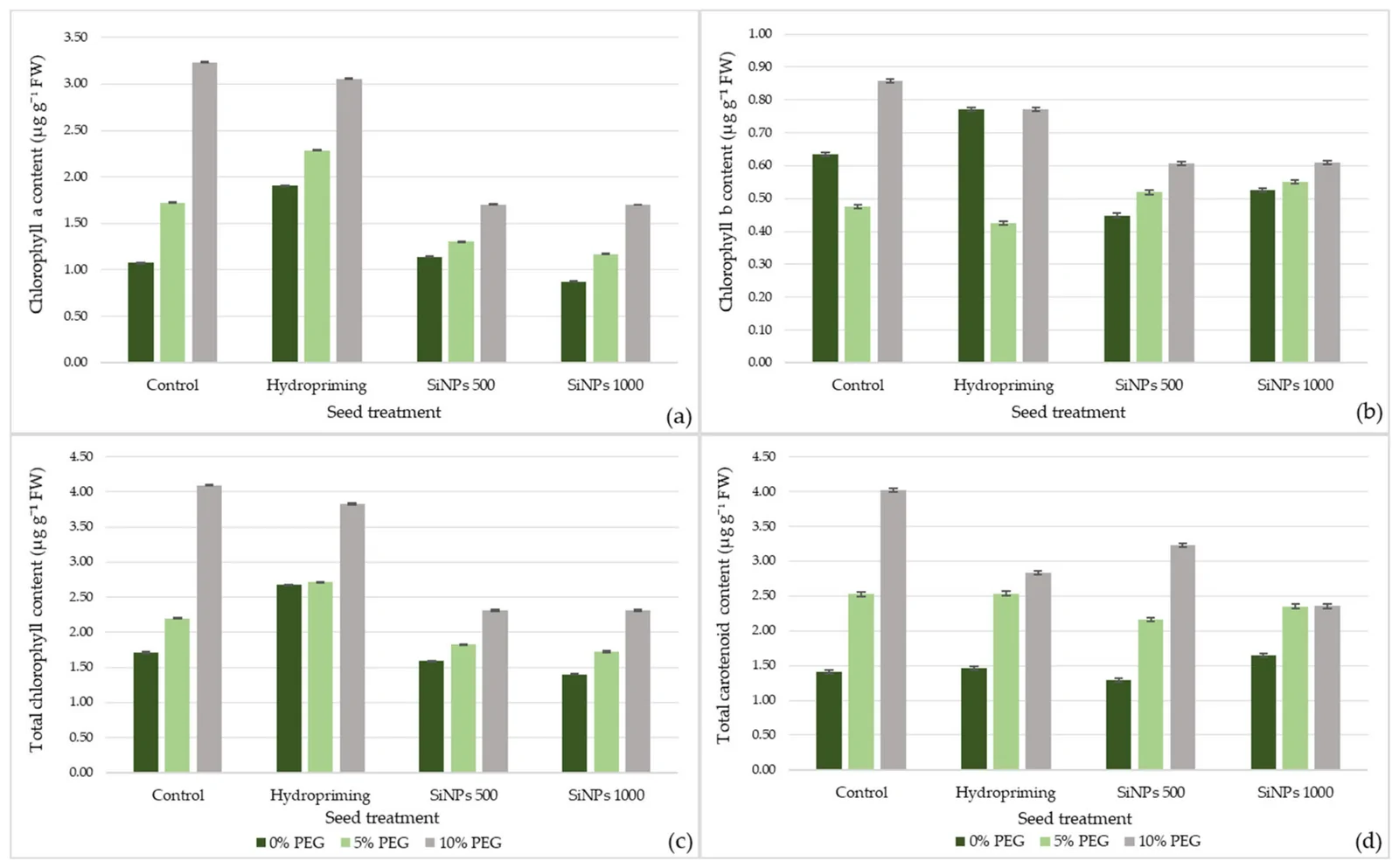
Effects of seed treatment on (**a**) chlorophyll a content (µg g^−1^ FW), (**b**) chlorophyll b content (µg g^−1^ FW), (**c**) total chlorophyll content (µg g^−1^ FW), and (**d**) total carotenoid content (µg g^−1^ FW) under different drought stress levels. Values represent means across 11 soybean genotypes for each combination of seed treatment and drought stress level. Bars represent mean values ± SE.

Photosynthetic pigment contents were significantly affected by seed treatment, drought stress, and genotype, with significant interactions among these factors (Table 3). Chlorophyll a content (CHL a) showed a treatment-dependent response across PEG levels (Figure 13a). Under non-stressed conditions and mild stress, hydropriming resulted in the highest mean values (1.9045 and 2.2869 µg g^−1^ FW, respectively), whereas under severe stress, the highest value was recorded in the untreated control (3.2361 µg g^−1^ FW). In contrast, both SiNP treatments showed substantially lower and nearly identical values under severe stress (1.7058 and 1.7016 µg g^−1^ FW for SiNPs 500 and SiNPs 1000, respectively).

Chlorophyll b (CHL b) showed a different response pattern (Figure 13b). Hydropriming resulted in the highest value under non-stressed conditions (0.7723 µg g^−1^ FW), whereas at 5% PEG, the highest values were recorded following SiNPs 1000 and SiNPs 500 (0.5502 and 0.5192 µg g^−1^ FW, respectively). At 10% PEG, the untreated control showed the highest value (0.8583 µg g^−1^ FW), while both SiNP treatments resulted in lower and similar values (0.6078–0.6099 µg g^−1^ FW).

Total chlorophyll content reflected the combined responses of chlorophyll a and b (Figure 13c). Hydropriming resulted in the highest mean values under 0 and 5% PEG (2.6767 and 2.7117 µg g^−1^ FW, respectively). Under 10% PEG, however, total chlorophyll increased to 4.0945 µg g^−1^ FW in the untreated control and 3.8292 µg g^−1^ FW following hydropriming, whereas both SiNP treatments showed substantially lower and nearly identical values (2.3115–2.3136 µg g^−1^ FW).

Total carotenoid content (TCAR) showed a clearer increase with increasing PEG concentration across seed treatments (Figure 13d). Mean values ranged from 1.2876 to 1.6479 µg g^−1^ FW under non-stressed conditions and increased to 2.1641–2.5367 µg g^−1^ FW at mild stress. Under severe stress, the highest value was recorded in the untreated control (4.0240 µg g^−1^ FW), followed by SiNPs 500 (3.2330 µg g^−1^ FW), hydropriming (2.8343 µg g^−1^ FW), and SiNPs 1000 (2.3540 µg g^−1^ FW).

#### 2.3.3. Total Amylolytic Activity

**Figure 14:**
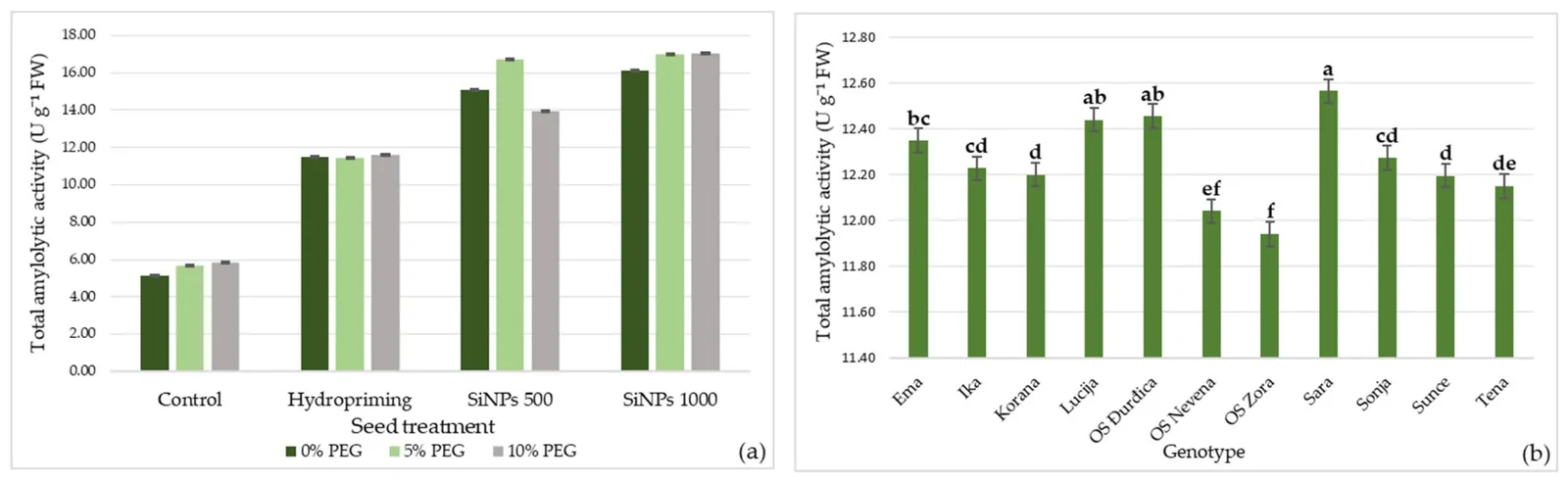
Effects of (**a**) seed treatment under different drought stress levels and (**b**) genotype on total amylolytic activity. In (**a**), values represent means across 11 soybean genotypes for each combination of seed treatment and drought stress level. In (**b**), values represent genotype means across all four seed treatments and three drought stress levels. Bars represent mean values ± SE. Different letters in (**b**) indicate statistically significant differences among genotypes according to the LSD test (*p* ≤ 0.05).

Total amylolytic activity (TAA) was consistently higher following seed priming than in the untreated control across all stress levels (Figure 14a). Hydropriming resulted in approximately twofold higher TAA than the untreated control, while both SiNP treatments showed a further increase in TAA. SiNPs 1000 generally showed the highest activity across stress levels, reaching 17.005 U g^−1^ FW at 5% PEG and 17.037 U g^−1^ FW under severe drought stress. SiNPs 500 increased TAA from 15.092 U g^−1^ FW at 0% PEG to 16.735 U g^−1^ FW at 5% PEG, followed by a decline to 13.922 U g^−1^ FW at 10% PEG. In contrast, TAA remained comparatively low in the untreated control, ranging from 5.110 to 5.843 U g^−1^ FW across stress levels. TAA also differed significantly among genotypes (Figure 14b), ranging from 11.942 U g^−1^ FW in *OS Zora* to 12.565 U g^−1^ FW in *Sara*. *Sara* showed the highest mean TAA, although it did not differ significantly from *Lucija* (12.440 U g^−1^ FW) and *OS Đurđica* (12.455 U g^−1^ FW), whereas *OS Zora* exhibited the lowest activity.

### 2.4. Correlation Analysis

Spearman’s correlation analysis revealed significant associations among germination, seedling growth, and biochemical traits, with the direction and strength of correlations varying according to seed treatment and stress level (Figures S3–S5). To emphasize the most pronounced relationships, only strong statistically significant correlations (|ρ| ≥ 0.70, *p* < 0.05) are highlighted below.

Under non-stressed conditions (0% PEG), strong correlations were largely treatment-specific (Figure S3a–d). In the untreated control, SFM was positively correlated with P (ρ = 0.745) and TF (ρ = 0.809), while P and TF were also positively correlated (ρ = 0.855). Following hydropriming, RL was positively correlated with GE (ρ = 0.843) and TPH (ρ = 0.827), while GE showed a particularly strong positive correlation with TPH (ρ = 0.938). Under SiNPs 500, negative correlations were observed between TAA and TF (ρ = −0.727) and between SL and TCAR (ρ = −0.709). Under SiNPs 1000, GE was positively correlated with P (ρ = 0.795), while NGS was negatively correlated with P (ρ = −0.822).

Under mild drought stress (5% PEG), the pattern of strong correlations shifted, with both positive and negative associations observed across seed treatments (Figure S4a–d). In the untreated control, RL was positively correlated with NS (ρ = 0.820) and TCHL (ρ = 0.774), while SFM was positively correlated with P (ρ = 0.782) and negatively correlated with TCAR (ρ = −0.873). Following hydropriming, TAA was positively correlated with SL (ρ = 0.818). Under SiNPs 500, RL was negatively correlated with TPH (ρ = −0.791), while TCHL was negatively correlated with AC (ρ = −0.800). Under SiNPs 1000, RL was strongly positively correlated with NS (ρ = 0.873) and positively correlated with TCHL (ρ = 0.727), while TCHL was positively correlated with TCAR (ρ = 0.755).

Under severe drought stress (10% PEG), strong associations became particularly evident among germination, seedling growth, and biochemical traits (Figure S5a–d). In the untreated control, GE showed a particularly strong positive correlation with TCAR (ρ = 0.909), while RL was positively correlated with GE (ρ = 0.791) and TCHL (ρ = 0.782). Under SiNPs 500, SFM was positively correlated with P (ρ = 0.827) and GE (ρ = 0.720), whereas SL was negatively correlated with NS (ρ = −0.709). Under SiNPs 1000, SFM was positively correlated with RL (ρ = 0.782), while RL was positively correlated with GE (ρ = 0.718) and P (ρ = 0.755) and negatively correlated with NGS (ρ = −0.706).

In general, the strongest correlations differed markedly among seed treatments and stress levels, indicating that the relationships among germination, seedling growth, and biochemical traits varied depending on experimental conditions.

## 3. Discussion

Recently, seed nanopriming has gained increasing attention as a strategy to improve seed performance and support early seedling growth under abiotic stress [25]. SiNP seed priming has been investigated in different crops, with reported effects on germination, early seedling growth, and physiological and biochemical responses under abiotic stress [36,37,38,39,40,42,43,44]. In the present study, the response of soybean seeds to SiNP priming varied in magnitude and direction depending on SiNP concentration, drought intensity, and genotype.

### 3.1. SiNP-Mediated Seed Priming Modulates Germination Responses to Drought Stress

As drought stress intensified, soybean germination was markedly impaired, with germination energy decreasing from 95.0% under non-stressed conditions to 72.2% at 10% PEG. Nevertheless, both SiNP treatments improved overall germination performance compared with untreated seeds, as reflected by higher germination energy and total germination and a lower proportion of non-germinated seeds. Hydropriming produced a comparable response, suggesting that part of the observed improvement may be attributed to the priming process itself. Controlled seed hydration initiates pre-germinative metabolic processes that facilitate subsequent germination [20]. Beyond this general priming effect, SiNPs may further modify seed responses during germination under water deficit. In wheat, Rai-Kalal et al. [36] reported enhanced germination following SiO_2_ nanopriming under drought stress, while Vanitha et al. [42] observed improved germination and seedling vigor in nano-silica-primed soybean seeds exposed to PEG-induced moisture stress.

However, an important distinction emerged between germination and the development of morphologically normal seedlings. Although priming increased total germination and reduced the proportion of non-germinated seeds, this was not accompanied by an overall increase in normal seedlings. In particular, SiNPs 500 resulted in a significantly lower mean proportion of normal seedlings and a significantly higher proportion of abnormal seedlings than the untreated control. This distinction is biologically relevant, as radicle emergence does not necessarily result in the subsequent development of a normal seedling [45]. Thus, while priming promoted germination, its effects on subsequent seedling development were more variable and depended on genotype, treatment, and drought stress intensity. The genotype-specific nature of the response was particularly evident under severe drought stress. Under 10% PEG, for example, germination energy in *Ika* was 13.0% in untreated seeds compared with 64.5% following SiNPs 1000, while SiNPs 500 maintained germination energy above 90% in *Ema* and *Lucija*. Similarly, the proportion of normal seedlings in *Ika* increased from 11.5% in untreated seeds to 40.0% following SiNPs 1000. Seed vigor showed a comparable pattern, with SiNP priming maintaining relatively high SVI in several genotypes under severe stress, including *Korana*, *Ika*, and *Ema*. However, this response was not universal, as SiNPs 1000 resulted in a lower SVI than the untreated control in *Tena*. These contrasting responses indicate that the effectiveness of SiNP priming cannot be generalized across soybean genotypes or explained by a simple concentration-dependent response. Rather, the effectiveness of different SiNP concentrations appears to depend on genotype and stress conditions. Genotype-dependent responses to nanopriming have also been reported in previous studies. Zulifqar et al. [46] observed varietal differences in germination and seedling growth responses to different nanoparticle types and concentrations in soybean, while Tamindžić et al. [47] reported cultivar-specific responses of germination and early seedling growth traits to nanopriming in field pea. Together, these findings emphasize the importance of considering genotype when optimizing nanoparticle-based seed priming treatments.

### 3.2. Early Seedling Growth Responses

Increasing drought stress had a more pronounced effect on early seedling growth than on germination-related traits. Total seedling length and seedling fresh mass progressively declined with increasing PEG concentration, with shoot elongation showing particularly high sensitivity. In contrast, root length was comparatively maintained, resulting in an increased contribution of roots to total seedling length and a higher root-to-shoot length ratio. Under severe drought stress (10% PEG), roots accounted for approximately 75–78% of total seedling length across seed treatments.

The pronounced inhibition of shoot elongation under increasing drought stress may be associated with the high sensitivity of cell expansion to reduced water availability. Decreased water potential limits water uptake and cellular turgor, thereby restricting cell expansion and elongation [48,49]. In contrast, root growth can be comparatively better maintained at low water potentials through mechanisms including osmotic adjustment, turgor maintenance, and changes in cell-wall properties [50]. A similar differential response was reported in soybean by Bukan et al. [15], who observed reductions in both root and shoot length under PEG-induced drought stress, with a smaller reduction in root length (11%) than in shoot length (17%), resulting in an increased root-to-shoot length ratio. Thus, water deficit may alter the relative development of roots and shoots rather than affecting both components of seedling growth to the same extent.

SiNP priming further modified this response in a genotype-dependent manner. Under severe drought stress, both SiNP treatments showed higher root-to-shoot ratios than the untreated control in *Ema*, *Ika*, *Korana*, *Lucija*, and *OS Đurđica*. This shift primarily reflected the greater inhibition of shoot elongation relative to root growth. The magnitude of growth inhibition also varied markedly depending on genotype and SiNP concentration. For example, *Tena* showed a 74.6% reduction in total seedling length between 0 and 10% PEG following SiNPs 1000 treatment. Overall, these genotype-specific patterns indicate that the effects of SiNP priming on early seedling growth depend on both nanoparticle concentration and genotype. Previous studies have similarly reported concentration-dependent effects of SiO_2_ nanopriming on seedling growth under drought stress. Rai-Kalal et al. [36] reported improved seedling performance following SiO_2_ nanopriming in drought-stressed wheat, while Raza et al. [39] further demonstrated that the magnitude of SiNP-mediated growth responses under drought varied among the concentrations tested. Together with the genotype-specific responses observed in the present study, these findings highlight the importance of both nanoparticle concentration and plant genotype in determining the effectiveness of SiNP seed priming under drought stress.

### 3.3. Proline Accumulation and Antioxidant Responses

One of the clearest biochemical responses observed in the present study was the progressive accumulation of proline with increasing drought intensity. This response was particularly pronounced following SiNP treatments, with SiNPs 1000 showing the highest mean proline content under severe drought stress, while substantial genotypic variation was also observed. Proline accumulation is a common response to water deficit and may contribute to osmotic adjustment and maintenance of cellular redox and ROS homeostasis [16]. The enhanced proline accumulation observed following SiNP priming under severe stress is consistent with Vanitha et al. [42], who reported increased proline accumulation in nano-silica-primed soybean under PEG-induced drought stress, together with improved relative water content and antioxidant enzyme activity.

Total phenolic and flavonoid contents showed pronounced variation among genotypes, treatments, and stress levels. Both are important components of the non-enzymatic antioxidant system and may contribute to cellular protection under abiotic stress [16]. Similarly, Eghlima et al. (2026) [51] report differences in the total phenol and flavonoid content among seven rose populations. Flavonoid content generally increased with increasing drought stress, although the magnitude of the response varied among treatments. Under severe drought stress, the highest flavonoid content was observed in untreated seedlings, closely followed by SiNPs 1000. In contrast, phenolic content showed a distinct treatment-dependent response to increasing drought stress: it decreased progressively in untreated seedlings, whereas SiNPs 1000 maintained relatively high phenolic content across stress levels. Under severe drought stress, SiNPs 1000 also showed a higher value than the untreated control, indicating a different pattern of phenolic response following SiNP priming as stress intensity increased.

Antioxidant capacity also showed a stress-dependent response. It was highest in untreated seedlings under non-stressed conditions, whereas the highest antioxidant capacity under severe drought stress was observed following SiNPs 1000, broadly paralleling the response of phenolic content. Unlike phenolic and flavonoid contents, however, antioxidant capacity showed no significant genotype-related interactions. Overall, these findings suggest that SiNP priming modulated the non-enzymatic antioxidant response in a stress-dependent manner rather than uniformly enhancing antioxidant capacity. A stress-dependent response of DPPH radical-scavenging activity to nanopriming has also been reported by Ochoa-Chaparro et al. [37].

### 3.4. Photosynthetic Pigment Responses

Photosynthetic pigments were included in the biochemical analyses to assess whether their contents responded to seed treatment and PEG-induced drought stress during early seedling development, rather than as indicators of photosynthetic performance. The observed changes in photosynthetic pigment contents should be interpreted in the context of the pronounced reduction in seedling fresh mass and changes in seedling morphology under drought stress. Because pigments were quantified on a fresh-weight basis (FW) using whole seedlings, including cotyledons, shoots, and roots, changes in the relative contribution of these tissues to total fresh mass may have influenced the measured pigment contents, without necessarily indicating greater total pigment accumulation. This is particularly relevant under severe drought stress, where shoot growth was strongly inhibited while root growth was comparatively maintained, substantially altering seedling morphology. Furthermore, because the seedlings were grown in darkness, these pigment responses should not be interpreted as direct evidence of enhanced or reduced photosynthetic capacity.

Within this context, pigment responses differed markedly among seed treatments and stress levels. Under severe drought stress, chlorophyll a, chlorophyll b, and total chlorophyll contents were highest in untreated seedlings, whereas both SiNP treatments showed substantially lower values. Carotenoid content showed a more consistent response to increasing drought stress, generally increasing with PEG concentration across seed treatments and reaching the highest level in untreated seedlings at 10% PEG. Given their important roles in photoprotection and plant stress responses, carotenoids may contribute to cellular protection under adverse environmental conditions [52]. In soybean, Esan et al. [53] reported genotype-dependent changes in carotenoid content under PEG-induced drought stress, with carotenoid content increasing in some genotypes but decreasing in others. Accordingly, the increases in carotenoid content observed with increasing drought stress may form part of the biochemical response to water deficit; however, as with chlorophylls, the fresh-weight basis of expression should be considered when interpreting these changes. Overall, the significant interactions among genotype, seed treatment, and stress level indicate that pigment responses were strongly dependent on experimental conditions rather than showing a uniform response to SiNP priming.

### 3.5. Total Amylolytic Activity and Reserve Mobilization

Total amylolytic activity (TAA) showed one of the clearest responses to seed priming, with activity consistently higher following priming than in the untreated control across all stress levels. Hydropriming showed higher TAA than the untreated control, while both SiNP treatments showed a further increase in TAA across all genotypes and PEG levels. Among the SiNP treatments, SiNPs 1000 generally showed the highest activity and maintained high TAA across increasing stress intensity, while SiNPs 500 showed a decline under severe drought stress. This pronounced priming response is consistent with the activation of pre-germinative metabolic processes during controlled seed hydration, including hydrolytic enzymes involved in reserve mobilization [20]. The particularly high TAA following SiNP priming may indicate an additional effect on hydrolytic processes beyond that induced by hydration alone. In wheat, Rai-Kalal et al. [36] similarly reported increased amylase activity following SiO_2_ nanopriming, together with enhanced seed water uptake, germination, seedling growth, and vigor. More recent evidence supports an effect of silica nanoparticle priming on seed reserve metabolism: silica-primed rice seeds showed enrichment of sugars and amino acids relative to hydroprimed seeds, which was associated with accelerated mobilization of stored energy reserves under PEG-induced drought stress [40]. The same study linked silica nanopriming to redox signaling and activation of drought-responsive genes, providing a possible signaling component that could contribute to metabolic activation beyond hydration alone [40]. Whether similar processes contribute to the increased TAA in soybean remains to be established. The physiological significance of this response should also be considered in the context of soybean carbohydrate metabolism. Germinating soybean seeds accumulate transient starch reserves, probably through gluconeogenesis from storage lipids, and possess α-amylase and α-glucosidase activities potentially involved in their subsequent mobilization [54]. Accordingly, the higher TAA following SiNP priming may reflect an increased capacity for starch turnover within the broader process of reserve utilization during early seedling development. Nevertheless, substantial differences in β-amylase activity among soybean cultivars have been reported without corresponding differences in starch metabolism, demonstrating that enzyme activity alone does not necessarily indicate the rate of starch mobilization in vivo [55]. Overall, these present findings suggest that enhanced starch-hydrolyzingcapacitymay represent one component of the SiNP priming response during soybean germination. Its occurrence under both non-stressed and PEG-induced drought stress conditions is consistent with a general metabolic response to priming. However, increased TAA alone does not establish a mechanism of drought tolerance or demonstrate accelerated reserve mobilization. Since the assay measures the combined starch-hydrolyzing capacity of the extract, further measurements of soluble carbohydrates (sugars) over time, together with specific amylase activity assays, would help clarify the metabolic consequences of the observed increase.

### 3.6. Associations Among Germination, Seedling Growth, and Biochemical Responses

Correlation analysis further showed that the relationships among germination, seedling growth, and biochemical traits varied according to seed treatment and drought stress intensity. Under severe drought stress, a particularly notable pattern was observed following SiNPs 1000, where seedling fresh mass showed a strong positive correlation with root length (ρ = 0.782), while root length was positively associated with germination energy (ρ = 0.718) and proline accumulation (ρ = 0.755) and negatively associated with the proportion of non-germinated seeds (ρ = −0.706). These relationships suggest that, under severe drought stress, greater root growth following SiNPs 1000 treatment was associated with higher germination performance and proline accumulation.

These relationships should, however, be interpreted as associations rather than evidence of causality. Proline accumulation may contribute to osmotic adjustment under drought stress; however, proline content alone should not be considered a direct indicator of drought tolerance, as proline metabolism and turnover also play important roles in plant responses to water limitation [56]. Similarly, associations among germination and growth traits may partly reflect the close relationship between these components of seed performance, which together contribute to seed vigor under adverse conditions [57]. Rather than any individual correlation, the marked shifts in correlation patterns among seed treatments and PEG levels therefore suggest that the relationships among germination, seedling growth, and biochemical responses varied depending on experimental conditions.

### 3.7. Study Limitations

A limitation of this study is that the SiO_2_ nanoparticles and prepared suspensions were not independently characterized beyond the manufacturer-reported specifications. In addition, because all biochemical parameters were expressed on a fresh-weight basis, PEG-induced differences in tissue water status may have influenced the magnitude of the measured biochemical responses. Finally, the present study was limited to germination and early seedling growth under controlled PEG-induced drought stress. Therefore, the observed responses should not be directly extrapolated to soil-based drought conditions, later developmental stages, or field performance. Further studies under soil-based and field conditions are needed to determine whether these early responses translate into improved agronomic performance under drought.

## 4. Materials and Methods

### 4.1. Seed Material

Seeds of 11 soybean genotypes (*Ema*, *Ika*, *Korana*, *Lucija*, *OS Đurđica*, *OS Nevena*, *OS Zora*, *Sara*, *Sonja*, *Sunce*, and *Tena*) developed by the Agricultural Institute Osijek, Osijek, Croatia, were used in this study. The seeds were produced in 2025 in production areas located in the vicinity of Osijek, Croatia (approximately 45°32′ N, 18°44′ E), at an elevation ranging from 88 to 90 m a.s.l. All analyses were performed at the Faculty of Agrobiotechnical Sciences Osijek (Osijek, Croatia) under controlled conditions.

### 4.2. Seed Sterilization Treatment

Before seed priming, soybean seeds were surface-sterilized by chlorine gas fumigation in a vacuum desiccator according to the method described by Šimić et al. [58]. This dry sterilization method was selected to avoid additional seed hydration and the potential cracking of the seed coat associated with liquid-based sterilization treatments.

### 4.3. Seed Priming Solutions

Silicon dioxide nanoparticles (SiO_2_ NPs; silicon dioxide nanopowder, particle size 10–20 nm (BET) and purity 99.5% trace metals basis, according to the manufacturer; Sigma-Aldrich, St. Louis, MO, USA) were dispersed in distilled water for 15 min using a UP400St ultrasonic homogenizer (Hielscher Ultrasonics, Teltow, Germany) to prepare suspensions at concentrations of 500 and 1000 mg L^−1^. The SiO_2_ NP concentrations were selected based on the soybean study of Vanitha et al. [42], in which 500 mg L^−1^ was identified as an effective concentration, while 1000 mg L^−1^ was included in the present study to assess the response to a higher SiNP concentration. Four seed treatments were applied: (1) untreated seeds (control), (2) hydropriming with distilled water (hydropriming), (3) priming with SiO_2_ NPs at 500 mg L^−1^ (SiNPs 500), and (4) priming with SiO_2_ NPs at 1000 mg L^−1^ (SiNPs 1000). Control seeds remained untreated and were not subjected to either preconditioning or priming.

To minimize potential damage to the seed coat, soybean seeds were subjected to a preconditioning step followed by priming with distilled water or the corresponding SiO_2_ nanoparticle suspension, following the approach described by Vanitha et al. [42]. Preconditioning was performed at a seed-to-solution ratio of 1:0.3 (*w*/*v*) for 2 h, followed by priming at a ratio of 1:1 (*w*/*v*) for an additional 2 h at room temperature. Following treatment, the seeds were removed from the solutions and dried at room temperature until a constant weight was reached before being used in the germination experiment. The SiO_2_ NP suspensions were used exclusively during seed priming and were not subsequently applied to the germination paper.

### 4.4. Preparation of PEG 6000 Solutions

During soybean seed germination, drought stress was simulated using polyethylene glycol 6000 (PEG 6000, Sigma-Aldrich, St. Louis, MO, USA) solutions at concentrations of 5% and 10% (*w*/*v*), representing mild and severe drought stress, respectively. These concentrations were selected based on previous studies on soybean that employed comparable PEG 6000 levels to induce water limitation during germination and early seedling development [59,60]. The selected concentrations provided two contrasting levels of PEG-induced stress while maintaining sufficient germination and seedling development for subsequent morphological and biochemical analyses.

### 4.5. Soybean Seed Sowing

Germination tests were conducted using the between-paper method in accordance with the International Rules for Seed Testing (ISTA) [61]. Four replicates of 50 randomly selected seeds were used for each genotype × treatment × stress level combination. Each replicate, consisting of 50 seeds germinated in a separate paper roll, was considered an independent experimental unit for a given genotype × seed treatment × PEG level combination. Untreated control seeds or dried primed seeds were placed between filter paper sheets (80 g m^−2^; 580 mm × 290 mm;Ahlstrom Germany GmbH, Bärenstein, Germany) moistened with 30 mL of distilled water for the non-stressed condition (0% PEG) or with 30 mL of 5% or 10% PEG solution to impose mild and severe drought stress, respectively. The seed-containing sheets were then rolled, placed in labeled plastic bags, and positioned vertically in an incubator (ICH260C, Memmert, Schwabach, Germany). Germination was conducted in darkness in a controlled-environment germination chamber maintained at 25 °C and 80% relative humidity. Each PEG level was assigned to a separate shelf within the chamber. Within each shelf, paper rolls representing different genotypes and seed treatments were randomly positioned. Each individual paper roll was assigned a unique numerical code to ensure correct identification throughout the experiment. Following the assessment of germination energy on day 5, the rolls were returned to randomly available positions within their respective PEG-level shelves, while the numerical codes ensured that each roll was correctly reassigned to the appropriate experimental group. Following the assessment of germination energy on the fifth day after sowing, an additional 5 mL of distilled water or the corresponding PEG solution was added to each roll according to treatment.

### 4.6. Germination and Seedling Growth Assessment

Germination energy (GE) was determined on the fifth day after sowing as the percentage of seeds exhibiting radicle protrusion of at least 1 mm, following the criterion described by Kering et al. [62].

On the eighth day after sowing, the final germination assessment was performed. Seedlings were classified as normal or abnormal according to the criteria of the International Rules for Seed Testing (ISTA) [46] and the percentages of normal seedlings (NS), abnormal seedlings (AS), and non-germinated seeds (NGS) were recorded. Total germination (TG) was calculated as the percentage of all germinated seeds, including normal and abnormal seedlings. The seed vigor index (SVI) was calculated using a modification of the approach described by Abdul-Baki and Anderson [63]:

SVI = Normal seedlings (%) × Mean total seedling length (cm)

To assess early seedling growth, 10 normal seedlings were randomly selected from each replicate on the eighth day. Total seedling length (TSL), root length (RL), shoot length (SL), and seedling fresh mass (SFM) were determined. Total seedling length was measured from the root tip to the base of the shoot, excluding the cotyledons. Root and shoot lengths were recorded separately. Fresh mass was determined immediately after removing excess surface moisture by weighing 10 randomly selected normal seedlings per replicate. The entire seedlings, including cotyledons, were weighed together, and fresh mass per seedling was subsequently calculated. Based on the measured parameters, the root-to-shoot length ratio (R/S = RL/SL), root proportion of total seedling length (R/TSL (%) = (RL/TSL) × 100), and shoot proportion of total seedling length (S/TSL (%) = (SL/TSL) × 100) were calculated.

Following the measurements, whole 8-day-old seedlings, including the cotyledons, from each replicate were collected, placed in separately labeled aluminum foil, and stored at −80 °C until further biochemical analyses.

### 4.7. Biochemical Analyses

#### 4.7.1. Preparation of Crude Extracts

Whole 8-day-old soybean seedlings, including the cotyledons, stored at −80 °C were ground to a fine, homogeneous powder using a pre-chilled laboratory mill while the tissue was maintained in a frozen state with liquid nitrogen. For the determination of proline content, total flavonoid content, antioxidant capacity, and total phenolic content, crude extracts were prepared from homogenized seedlings using 80% (*v*/*v*) ethanol at a plant material-to-solvent ratio of 1:10 (*w*/*v*). Extraction was performed on an orbital shaker at 110 rpm for 60 min at room temperature, after which the samples were centrifuged at 20,000× *g* for 15 min at 4 °C. The resulting clear supernatants were carefully collected without disturbing the pellet and used for the spectrophotometric analyses. For the determination of photosynthetic pigment content, homogenized tissue was extracted with pure acetone at a plant material-to-solvent ratio of 1:2 (*w*/*v*). After the addition of acetone, the suspension was vortexed for 5 min and subsequently centrifuged at 20,000× *g* for 15 min. The resulting supernatant was used for spectrophotometric measurements. For the analysis of total amylolytic activity, homogenized plant material was extracted in cold 50 mM sodium acetate buffer (pH 5.5) containing 5 mM CaCl_2_, corresponding to a plant material-to-extraction buffer ratio of 1:5 (*w*/*v*). The suspensions were vortexed for 20 s at 10 min intervals and extracted at 4 °C for 30 min. After extraction, the samples were centrifuged at 20,000× *g* for 15 min at 4 °C, and the resulting supernatants were used as crude enzyme extracts.

#### 4.7.2. Proline Content Determination

Proline content (P) was determined using the ninhydrin colorimetric method described by Bates et al. [64], with modifications. Briefly, an aliquot (500 µL) of the diluted extract was mixed with 1.0 mL of 1% (*w*/*v*) ninhydrin reagent prepared in a solution containing 60% (*v*/*v*) acetic acid and 20% (*v*/*v*) ethanol. The reaction mixture was vortexed and incubated at 95 °C for 20 min, cooled to room temperature, and centrifuged at 5000× *g* for 1 min. Absorbance was measured at 520 nm against the corresponding blank. Proline content was calculated using an L-proline calibration curve (0–500 nmol), and expressed as µmol proline g^−1^ fresh weight.

#### 4.7.3. Total Flavonoid Content Determination

Total flavonoid content (TF) was determined using the aluminum chloride colorimetric method described by Valdés et al. [65], with minor modifications. An aliquot (125 µL) of the ethanolic extract was mixed with an equal volume of 2% (*w*/*v*) AlCl_3_ solution in a 96-well microplate. The reaction mixture was incubated for 30 min at room temperature in the dark, after which absorbance was measured at 415 nm. Quercetin was used as the reference standard, and the results were calculated from the corresponding calibration curve of quercetin (0–1.5 µg) and expressed as micrograms of quercetin equivalents per gram of fresh weight (µg QE g^−1^ FW).

#### 4.7.4. Assessment of Antioxidant Capacity

The antioxidant capacity (AC) was assessed using the 2,2-diphenyl-1-picrylhydrazyl (DPPH) radical-scavenging assay according to Brand-Williams et al. [66], with modifications for a 96-well microplate. The diluted extract was prepared by mixing 20 µL of the original extract with 105 µL of 80% ethanol and 125 µL of freshly prepared 100 µM DPPH solution. The DPPH control contained 125 µL of 80% ethanol and 125 µL of the DPPH solution. A corresponding sample blank, consisting of 20 µL of the original extract and 230 µL of 80% ethanol without DPPH, was used to correct for the absorbance of the extract. 6-hydroxy-2,5,7,8-tetramethylchroman-2-carboxylic acid (Trolox) was used as the antioxidant standard. The reaction mixtures were incubated for 30 min at room temperature, protected from light, after which absorbance was measured at 517 nm. DPPH radical inhibition was calculated according to the following equation:

DPPH inhibition (%) = [(Acontrol − Asample,corr)/Acontrol] × 100

where Acontrol is the absorbance of the DPPH control and Asample,corr is the sample absorbance corrected using the corresponding sample blank. The antioxidant capacity was determined from the Trolox calibration curve (0–10 nmol) and expressed as µmol Trolox equivalent antioxidant capacity per gram of fresh weight (µmol TEAC g^−1^ FW).

#### 4.7.5. Total Phenolic Content Determination

Total phenolic content (TPH) was determined using the Fast Blue BB (FBBB) method with 4-benzoylamino-2,5-diethoxybenzenediazonium chloride hemi (zinc chloride) salt, according to Medina [67], in a 96-well microplate. An aliquot of 50 µL of the 80% ethanolic extract was pipetted into each well, and the volume was adjusted to 150 µL with 80% ethanol. Subsequently, 50 µL of freshly prepared 0.05% (*w*/*v*) FBBB reagent in 80% ethanol was added. The contents of each well were mixed, followed by the addition of 50 µL of 2% (*w*/*v*) NaOH, and the reaction mixture was mixed again. The microplate was incubated for 60 min at room temperature, protected from light, after which the absorbance was measured at 420 nm. Quantification was performed using a gallic acid calibration curve (0–12.5 µg) prepared in 80% ethanol and analyzed under the same reaction conditions as the samples. The results were expressed as milligrams of gallic acid equivalents per gram of fresh weight (mg GAE g^−1^ FW).

#### 4.7.6. Photosynthetic Pigment Quantification

The content of chlorophyll a (CHL a), chlorophyll b (CHL b), total chlorophyll (TCHL), and total carotenoid (TCAR) was quantified using the equations for pure acetone described by Lichtenthaler and Wellburn [68]. A 1 mL aliquot of the supernatant was used to measure the absorbance at 470, 645, and 662 nm, with pure acetone serving as a blank. The obtained values were converted based on the extract volume and the mass of the extracted plant material, and the results were expressed as µg g^−1^ FW.

#### 4.7.7. Assessment of Total Amylolytic Activity

Total amylolytic activity (TAA) was assessed using the method of Bernfeld [69], based on the spectrophotometric quantification of reducing sugars with 3,5-dinitrosalicylic acid (DNS). Before analysis, the crude enzyme extracts were appropriately diluted with the extraction buffer. The reaction mixture consisted of 250 µL of 1% (*w*/*v*) soluble starch prepared in 50 mM sodium acetate buffer (pH 5.5) containing 5 mM CaCl_2_ and 250 µL of the diluted enzyme extract. The reaction mixture was mixed and incubated at 37 °C for exactly 10 min. The reaction was terminated by adding 500 µL of 40 mM DNS reagent and the samples were heated in a boiling water bath for 5 min, cooled to room temperature, and diluted with 5.00 mL of purified water. Absorbance was measured at 540 nm. A corresponding sample blank was prepared for each enzyme extract and contained 250 µL of the diluted enzyme extract, 500 µL of 40 mM DNS reagent, and 250 µL of 1% (*w*/*v*) soluble starch. In the sample blanks, the DNS reagent was added to the enzyme extract before the addition of starch to inactivate the enzymes before substrate hydrolysis could occur. The amount of reducing sugars released during starch hydrolysis was determined using a maltose calibration curve (0–2.50 µmol) and expressed as maltose equivalents. One unit of total amylolytic activity (U) was defined as the amount of enzyme activity that released 1 µmol of reducing groups, expressed as maltose equivalents, per minute under the assay conditions. Total amylolytic activity was expressed per gram of seedling fresh weight (U g^−1^ FW).

### 4.8. Statistical Analysis

Three-way analysis of variance (three-way ANOVA) was performed using SAS Enterprise Guide 7.1 to evaluate the effects of seed treatment (T), PEG-induced drought stress level (PEG), genotype (G), and their interactions (T × PEG, T × G, PEG × G, and T × PEG × G) on the measured parameters.

For percentage-based germination traits, percentages were calculated separately for each experimental replicate consisting of 50 seeds, and these replicate-level values were used in the analysis. Because the experiment had a balanced factorial design with equal replication across all genotype × treatment × PEG combinations, conventional ANOVA was used to evaluate the main effects and interactions.

For seedling growth traits, measurements from the 10 normal seedlings sampled within each replicate were averaged before statistical analysis, resulting in one replicate-level value per experimental unit. When a statistically significant F-test was detected, mean separation was performed using Fisher’s protected least significant difference (LSD) test at *p* ≤ 0.05. The LSD procedure was therefore applied only following a significant omnibus F-test and was used primarily for comparisons of significant main effects. Complex three-way interactions were evaluated from the factorial ANOVA and visualized using heat maps rather than subjected to exhaustive pairwise comparisons among all factor combinations.

Three-way interactions among seed treatment, PEG level, and genotype were visualized using heat maps generated with the ChiPlot online platform (version 2.6.1) [70]. Spearman’s rank correlation coefficients were calculated using R software (version 4.6.1; R Core Team, Vienna, Austria) [71] within the RStudio integrated development environment (version 2026.08.1, Build 195; Posit Software, PBC, Boston, MA, USA) [72]. Correlations were considered statistically significant at *p* < 0.05.

## 5. Conclusions

This study demonstrated that SiNP seed priming modulated soybean responses to PEG-induced drought stress during germination and early seedling growth. SiNP priming generally improved germination performance, whereas its effects on normal seedling development and growth were more variable. Increasing drought stress strongly inhibited seedling growth, particularly shoot elongation and seedling fresh mass, while root growth was comparatively maintained. SiNP priming also modified biochemical responses to PEG-induced drought stress, with particularly pronounced effects on proline accumulation and total amylolytic activity.

Importantly, the effects of SiNP priming varied depending on nanoparticle concentration, drought stress intensity, and genotype. These findings indicate that SiNP seed priming should be considered a genotype- and stress-dependent approach rather than a universally beneficial treatment. Overall, the results highlight the potential of SiNP seed priming to modulate early soybean responses to PEG-induced drought stress and emphasize the importance of considering nanoparticle concentration, genotype, and stress intensity when evaluating its effectiveness.

## Figures and Tables

**Table 1:** Summary of the three-way analysis of variance (ANOVA) for soybean seed germination traits.

Variation Source	*df*	TG	GE	NS	AS	NGS	SVI
*G*	10	58.10 ***	53.90 ***	39.42 ***	44.85 ***	58.10 ***	40.35 ***
*PEG*	2	329.50 ***	941.37 ***	360.68 ***	102.64 ***	329.50 ***	975.75 ***
*T*	3	37.37 ***	174.69 ***	5.21 **	35.25 ***	37.37 ***	59.73 ***
*T × PEG*	6	44.38 ***	117.88 ***	39.02 ***	11.14 ***	44.38 ***	15.30 ***
*T × G*	30	9.94 ***	5.70 ***	8.40 ***	7.45 ***	9.94 ***	9.75 ***
*PEG × G*	20	17.55 ***	18.10 ***	8.79 ***	3.34 ***	17.55 ***	7.66 ***
*T × PEG × G*	60	6.51 ***	4.45 ***	4.00 ***	3.93 ***	6.51 ***	5.81 ***

Note: *df*: degrees of freedom; TG: total germination; GE: germination energy; NS: percentage of normal seedlings; AS: percentage of abnormal seedlings; NGS: percentage of non-germinated seeds; SVI: seed vigor index. Numbers represent F-values. ** and *** indicate significance at *p* ≤ 0.01 and *p* ≤ 0.001, respectively.

**Table 2:** Summary of the three-way analysis of variance (ANOVA) for soybean seedling growth and morphological parameters.

Variation Source	*df*	SFM	TSL	RL	SL	R/S	R/TSL	S/TSL
*G*	10	89.75 ***	36.03 ***	40.49 ***	23.30 ***	16.27 ***	41.70 ***	41.70 ***
*PEG*	2	3968.90 ***	1433.47 ***	17.70 ***	5522.70 ***	825.51 ***	2028.91 ***	2028.91 ***
*T*	3	180.96 ***	213.86 ***	38.91 ***	431.21 ***	13.68 ***	61.74 ***	61.74 ***
*T × PEG*	6	51.99 ***	68.42 ***	15.00 **	223.49 ***	6.18 ***	51.24 ***	51.24 ***
*T × G*	30	2.59 ***	6.08 ***	5.72 ***	4.90 ***	4.23 ***	4.90 ***	4.90 ***
*PEG × G*	20	1.79 *	4.5 ***	5.22 ***	4.72 ***	3.17 ***	5.56 ***	5.56 ***
*T × PEG × G*	60	1.55 **	5.94 ***	6.2 ***	5.07 ***	5.86 ***	7.64 ***	7.64 ***

Note: *df*: degrees of freedom; SFM: seedling fresh mass; TSL: total seedling length; RL: root length; SL: shoot length; R/S: root-to-shoot length ratio; R/TSL: proportion of root in total seedling length; S/TSL: proportion of shoot in total seedling length. Numbers represent F-values. *, **, and *** indicate significance at *p* ≤ 0.05, *p* ≤ 0.01, and *p* ≤ 0.001, respectively.

**Table 3:** Summary of the three-way analysis of variance (ANOVA) for biochemical traits in soybean seedlings.

Variation Source	*df*	P	TF	AC	TPH	CHL a	CHL b	TCHL	TCAR	TAA
*G*	10	312.2 ***	1087.5 ***	3.0 **	47.4 ***	5665.4 ***	147.3 ***	1673.1 ***	46.2 ***	12.5 ***
*PEG*	2	7522.3 ***	9149.6 ***	10.10 ***	30.5 ***	93,119.3 ***	1386.8 ***	26,182.2 ***	3430.6 ***	215.6 ***
*T*	3	6577.4 ***	1227.4 ***	77.8 ***	1290.6 ***	57,780.2 ***	382.8 ***	15,583.5 ***	201.2 ***	25,022.0 ***
*T × PEG*	6	77.3 ***	160.5 ***	10.2 ***	128.8 ***	8326.9 ***	355.5 ***	2367.0 ***	242.1 ***	197.1 ***
*T × G*	30	30.8 ***	73.7 ***	0.9 ns	10.6 ***	1331.5 ***	272.4 ***	681.33 ***	13.6 ***	8.7 ***
*PEG × G*	20	30.6 ***	86.1 ***	0.9 ns	10.4 ***	1269.7 ***	178.7 ***	593.0 ***	18.7 ***	6.0 ***
*T × PEG × G*	60	23.7 ***	39.9 ***	0.6 ns	7.1 ***	966.1 ***	266.6 ***	556.2 ***	20.0 ***	5.8 ***

Note: *df*: degrees of freedom; P: proline content; TF: total flavonoid content; AC: antioxidant capacity; TPH: total phenolic content; CHL a: chlorophyll a content; CHL b: chlorophyll b content; TCHL: total chlorophyll content; TCAR: total carotenoid content; TAA: total amylolytic activity. Numbers represent F-values. ns indicates no significant difference (*p* > 0.05), while ** and *** indicate significance at *p* ≤ 0.01 and *p* ≤ 0.001, respectively.

## Data Availability

The data presented in this study are available from the corresponding author(s) upon reasonable request.

## Supplementary Materials

The following supporting information can be downloaded at: https://www.mdpi.com/article/10.3390/plants15203226/s1, Figure S1: Interaction between soybean genotype and seed treatment for percentage of normal seedlings (%) under (a) non-stressed conditions (0% PEG), (b) mild drought stress (5% PEG), and (c) severe drought stress (10% PEG). Values represent mean percentage of normal seedlings; Figure S2: Effects of seed treatment under different drought stress levels on (a) shoot length (cm), (b) root length (cm), (c) root proportion in total seedling length (%), and (d) shoot proportion in total seedling length (%). Values represent means across 11 soybean genotypes for each combination of seed treatment and drought stress level. Bars represent mean values ± SE; Figure S3: Spearman’s rank correlation matrices among germination, seedling growth, and biochemical traits across 11 soybean genotypes under non-stressed conditions (0% PEG) following different seed treatments: (a) control; (b) hydropriming; (c) SiNPs 500; and (d) SiNPs 1000. Statistically significant correlations (*p* < 0.05) are marked with an asterisk (*). The size and color of the squares represent the strength and direction of Spearman’s correlation coefficient (ρ), respectively, with green indicating positive and red indicating negative correlations; Figure S4: Spearman’s rank correlation matrices among germination, seedling growth, and biochemical traits across 11 soybean genotypes under mild drought stress conditions (5% PEG) following different seed treatments: (a) control; (b) hydropriming; (c) SiNPs 500; and (d) SiNPs 1000. Statistically significant correlations (*p* < 0.05) are marked with an asterisk (*). The size and color of the squares represent the strength and direction of Spearman’s correlation coefficient (ρ), respectively, with green indicating positive and red indicating negative correlations; Figure S5: Spearman’s rank correlation matrices among germination, seedling growth, and biochemical traits across 11 soybean genotypes under severe drought stress conditions (10% PEG) following different seed treatments: (a) control; (b) hydropriming; (c) SiNPs 500; and (d) SiNPs 1000. Statistically significant correlations (*p* < 0.05) are marked with an asterisk (*). The size and color of the squares represent the strength and direction of Spearman’s correlation coefficient (ρ), respectively, with green indicating positive and red indicating negative correlations.
