# Supplementary material for: Silicon Nanoparticle Seed Priming Modulates Germination, Early Seedling Growth, and Biochemical Responses of Soybean Under PEG-Induced Drought Stress
Source: Plants (Basel). 2026 Oct 21;15(20):3226. doi: 10.3390/plants15203226 (PMC13708790; doi:10.3390/plants15203226)
Supplement: Supplementary file 1 [file plants-15-03226-s001.zip › plants-4600323-supplementary.pdf]

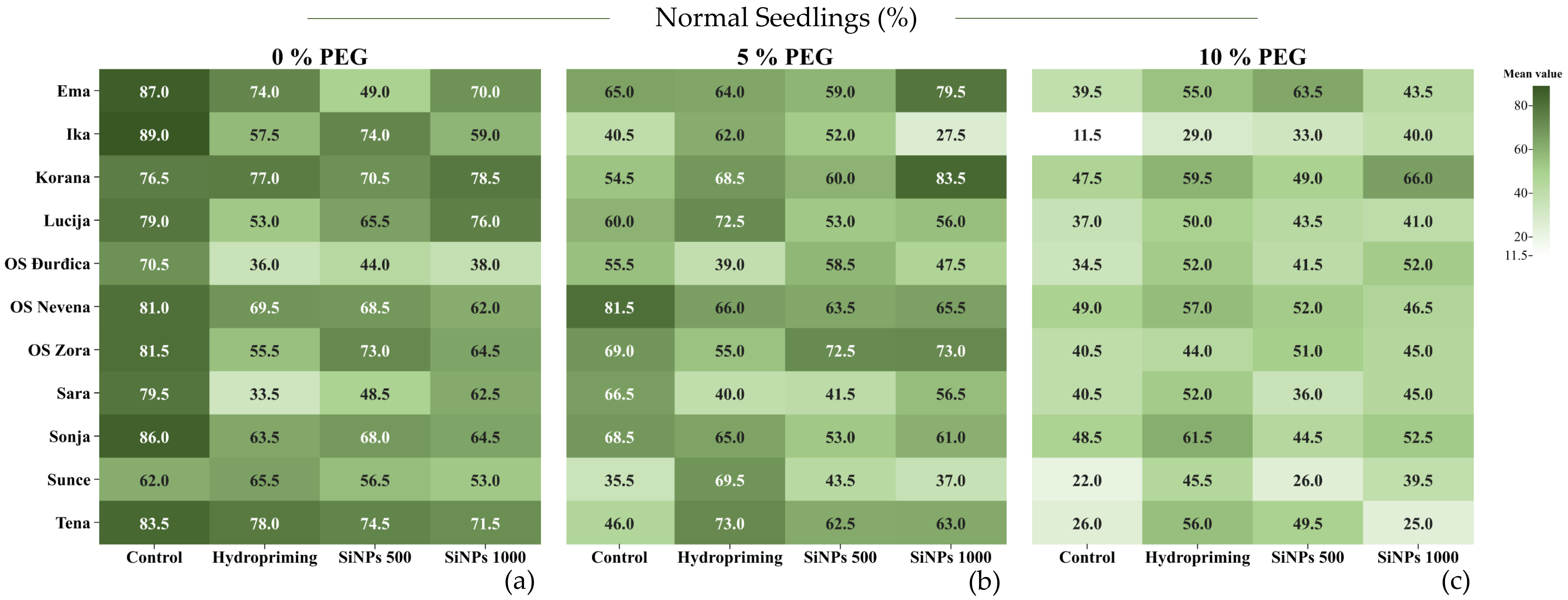

Figure S1. Interaction between soybean genotype and seed treatment for percentage of normal seedlings (%) under (a) non-stressed conditions (0% PEG), (b) mild drought stress (5% PEG), and (c) severe drought stress (10% PEG). Values represent mean percentage of normal seedlings.

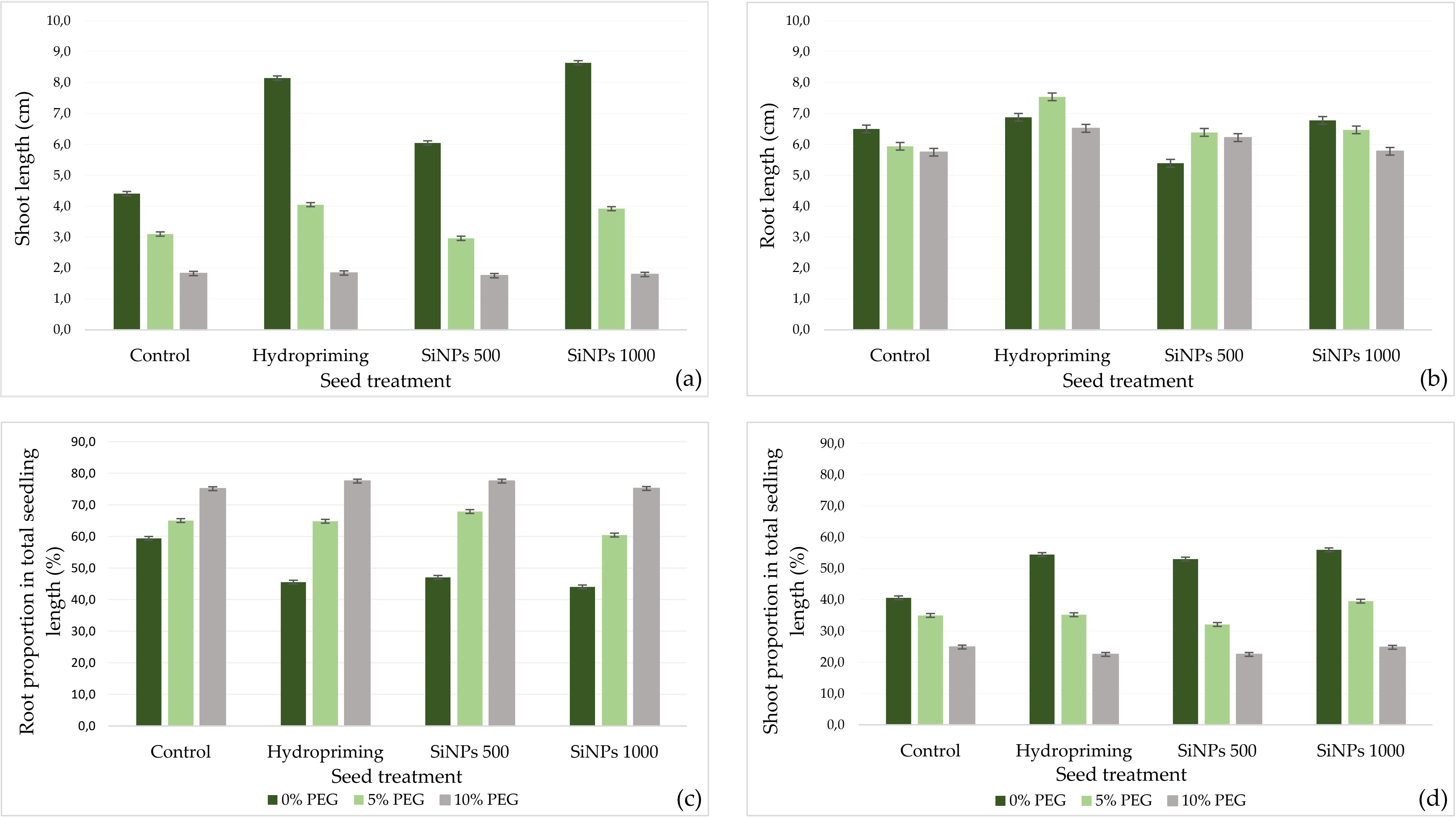

Figure S2. Effects of seed treatment under different drought stress levels on (a) shoot length (cm), (b) root length (cm), (c) root proportion in total seedling length (%), and (d) shoot proportion in total seedling length (%). Values represent means across 11 soybean genotypes for each combination of seed treatment and drought stress level. Bars represent mean values ± SE.

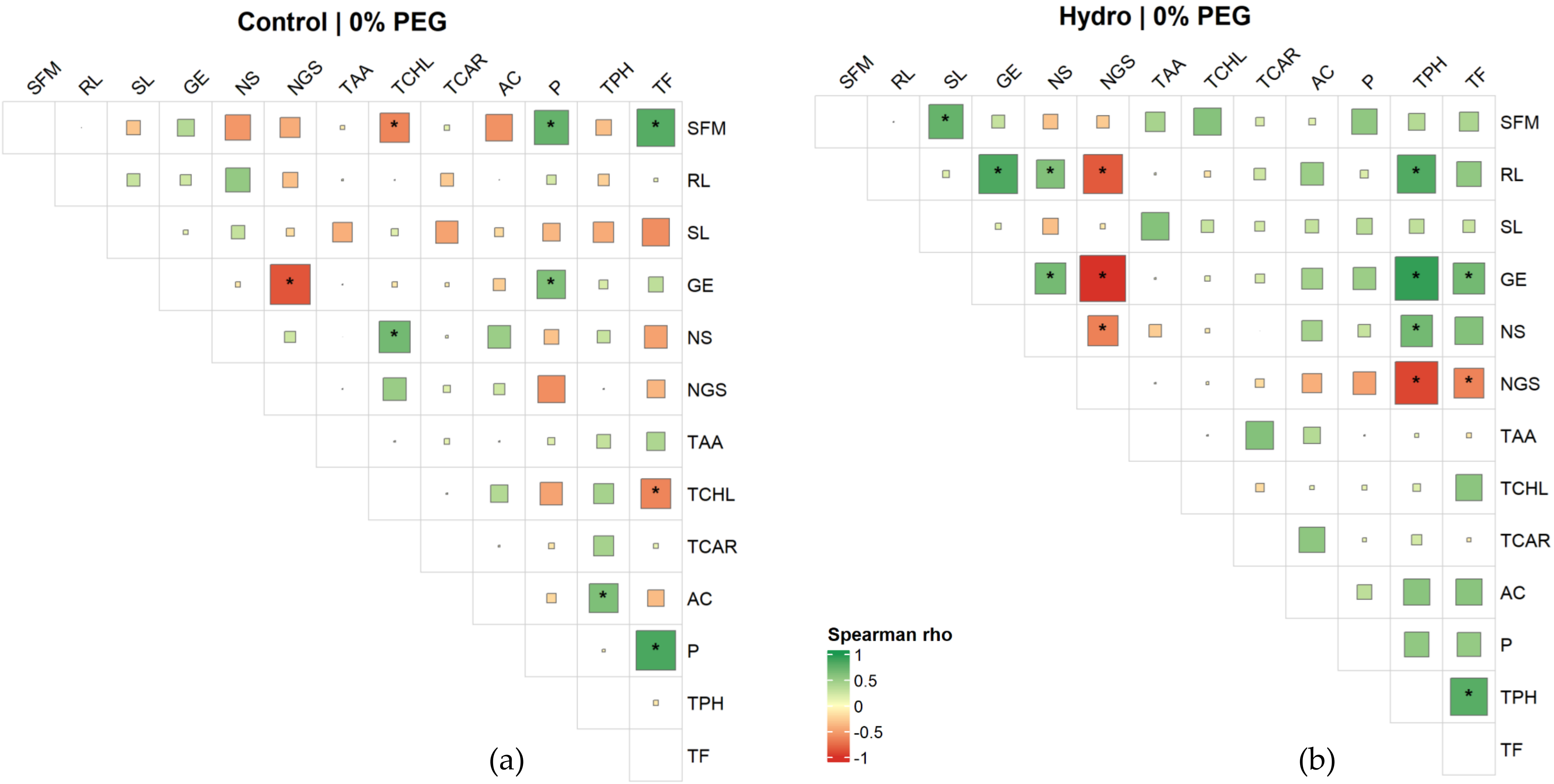
Control | 0% PEG
SFM RL SL GE NS NGS TAA TCHL TCAR AC P TPH TF
SFM RL SL GE NS NGS TAA TCHL TCAR AC P TPH TF
(a)
Hydro | 0% PEG
SFM RL SL GE NS NGS TAA TCHL TCAR AC P TPH TF
SFM RL SL GE NS NGS TAA TCHL TCAR AC P TPH TF
(b)
Spearman rho
1
0.5
0
-0.5
-1

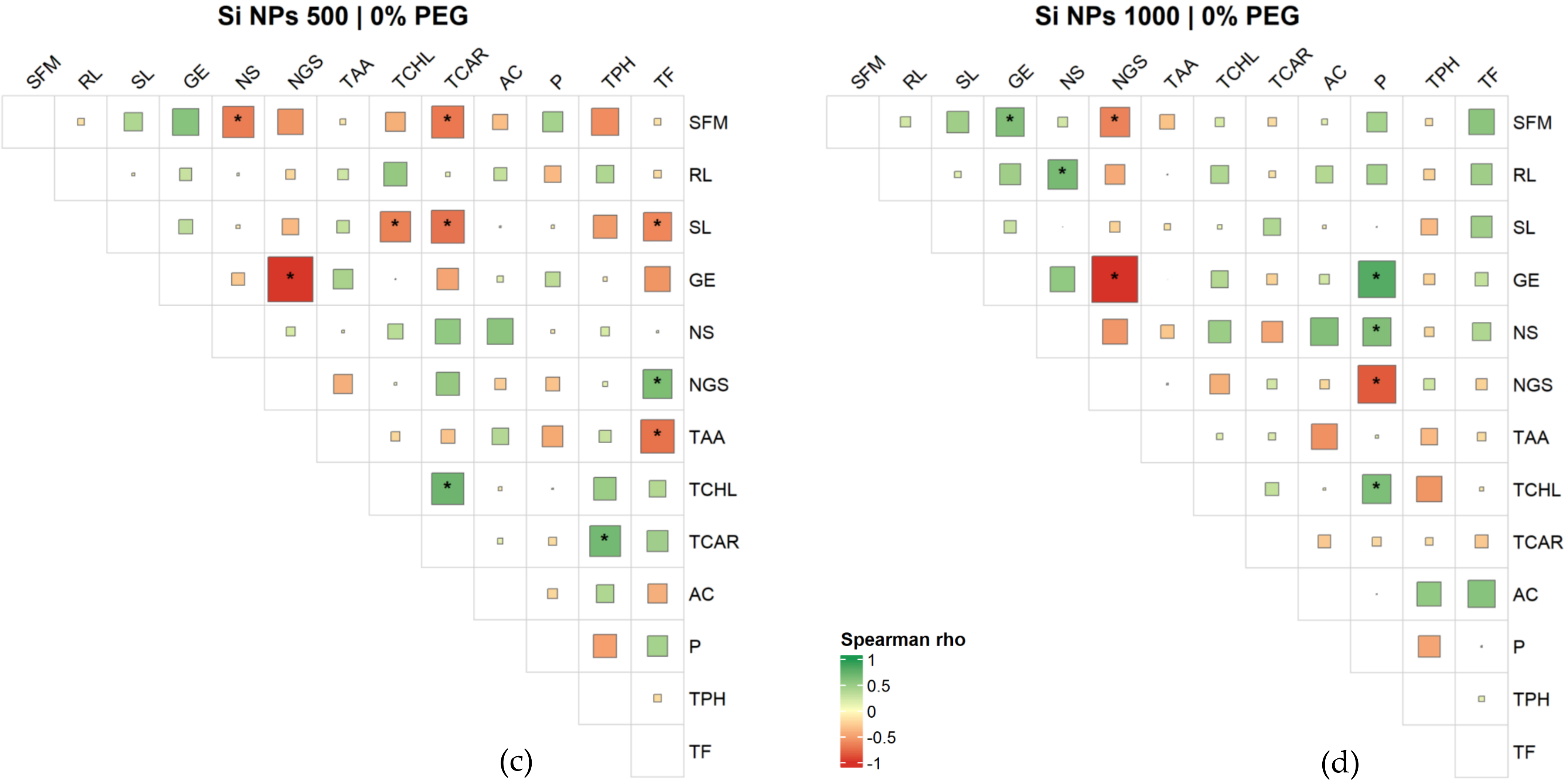

(c) (d)

Figure S3. Spearman's rank correlation matrices among germination, seedling growth, and biochemical traits across 11 soybean genotypes under non-stressed conditions (0% PEG) following different seed treatments: (a) control; (b) hydropriming; (c) SiNPs 500; and (d) SiNPs 1000. Statistically significant correlations ($p < 0.05$) are marked with an asterisk (*). The size and color of the squares represent the strength and direction of Spearman's correlation coefficient (ρ), respectively, with green indicating positive and red indicating negative correlations.

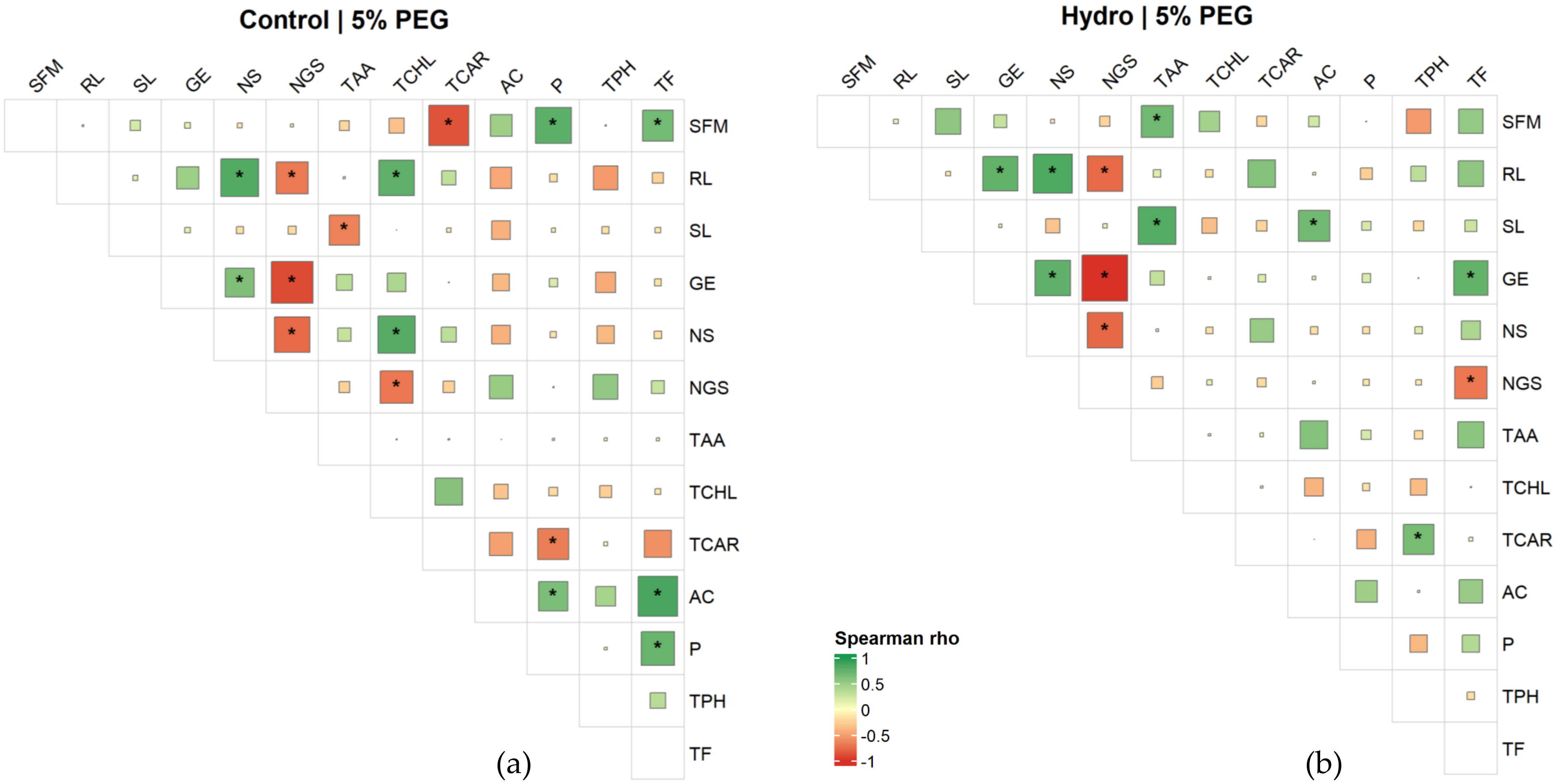

Control | 5% PEG
SFM RL SL GE NS NGS TAA TCHL TCAR AC P TPH TF
(a)
Hydro | 5% PEG
SFM RL SL GE NS NGS TAA TCHL TCAR AC P TPH TF
(b)
Spearman rho
1
0.5
0
-0.5
-1

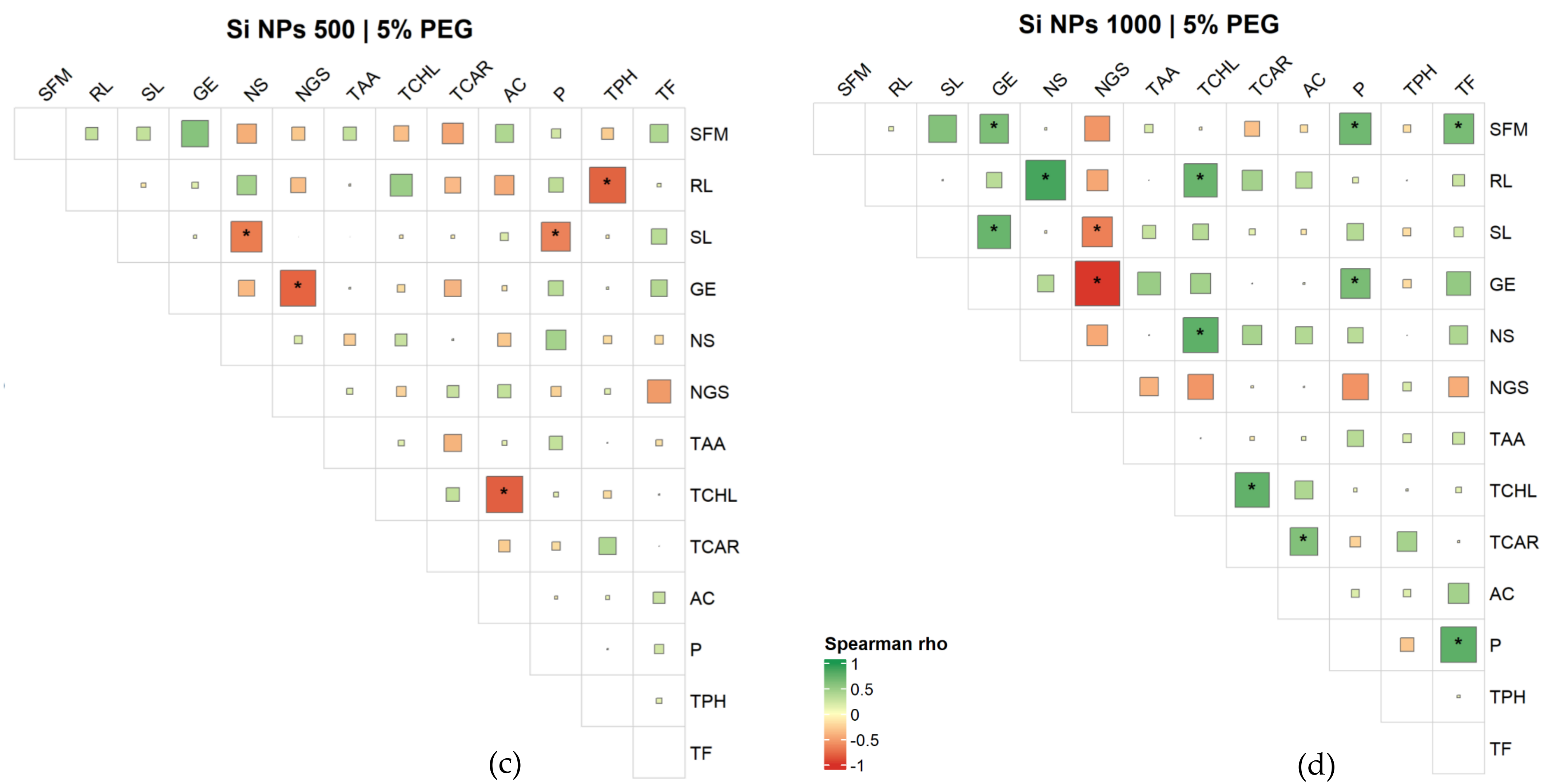

Figure S4. Spearman's rank correlation matrices among germination, seedling growth, and biochemical traits across 11 soybean genotypes under mild drought stress conditions (5% PEG) following different seed treatments: (a) control; (b) hydropriming; (c) SiNPs 500; and (d) SiNPs 1000. Statistically significant correlations ($p < 0.05$) are marked with an asterisk (*). The size and color of the squares represent the strength and direction of Spearman's correlation coefficient (ρ), respectively, with green indicating positive and red indicating negative correlations.

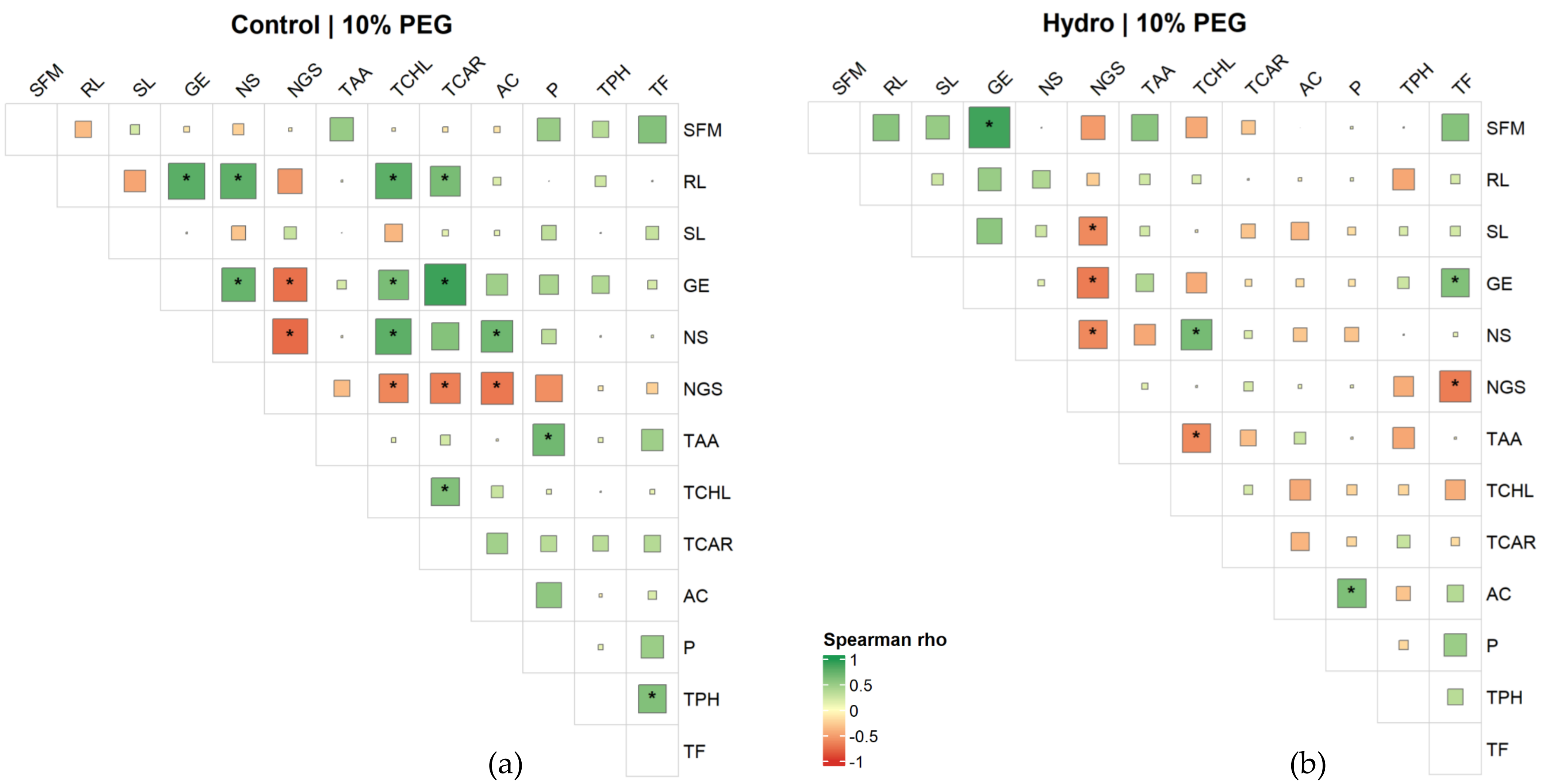
Control | 10% PEG
Hydro | 10% PEG
SFM
RL
SL
GE
NS
NGS
TAA
TCHL
TCAR
AC
P
TPH
TF
Spearman rho
1
0.5
0
-0.5
-1
(a)
(b)

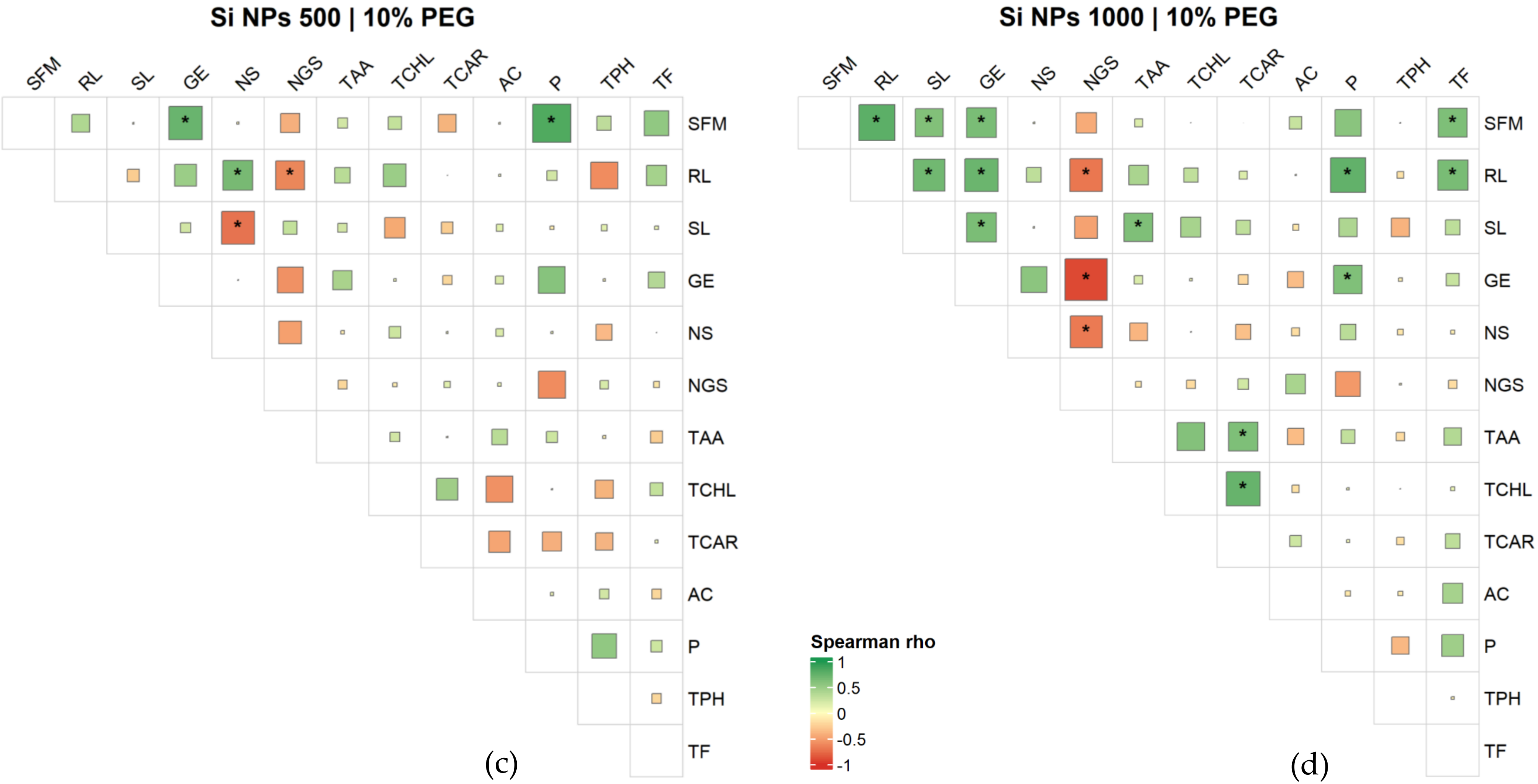

Figure S5. Spearman's rank correlation matrices among germination, seedling growth, and biochemical traits across 11 soybean genotypes under severe drought stress conditions (10% PEG) following different seed treatments: (a) control; (b) hydropriming; (c) SiNPs 500; and (d) SiNPs 1000. Statistically significant correlations ($p < 0.05$) are marked with an asterisk (*). The size and color of the squares represent the strength and direction of Spearman's correlation coefficient (ρ), respectively, with green indicating positive and red indicating negative correlations.
